# FXYD2 antisense oligonucleotide provides an efficient approach for long-lasting relief of chronic peripheral pain

**DOI:** 10.1172/jci.insight.161246

**Published:** 2023-05-08

**Authors:** Alexandre Derre, Noelian Soler, Valentine Billoux, Sebastien Benizri, Brune Vialet, Cyril Rivat, Philippe Barthélémy, Patrick Carroll, Alexandre Pattyn, Stephanie Venteo

**Affiliations:** 1 Institute for Neurosciences of Montpellier, University of Montpellier, INSERM, Montpellier, France.; 2 ARNA Laboratory, University of Bordeaux, INSERM U1212, UMR CNRS 5320, Bordeaux, France.

**Keywords:** Neuroscience, Pain

## Abstract

Chronic pain, whether of inflammatory or neuropathic origin, affects about 18% of the population of developed countries, and most current treatments are only moderately effective and/or cause serious side effects. Therefore, the development of novel therapeutic approaches still represents a major challenge. The Na,K-ATPase modulator FXYD2 is critically required for the maintenance of neuropathic pain in rodents. Here, we set up a therapeutic protocol based on the use of chemically modified antisense oligonucleotides (ASOs) to inhibit FXYD2 expression and treat chronic pain. We identified an ASO targeting a 20-nucleotide stretch in the *FXYD2* mRNA that is evolutionarily conserved between rats and humans and is a potent inhibitor of FXYD2 expression. We used this sequence to synthesize lipid-modified forms of ASO (FXYD2-LASO) to facilitate their entry into dorsal root ganglia neurons. We established that intrathecal or intravenous injections of FXYD2-LASO in rat models of neuropathic or inflammatory pain led to a virtually complete alleviation of their pain symptoms, without causing obvious side effects. Remarkably, by using 2′-*O*-2-methoxyethyl chemical stabilization of the ASO (FXYD2-LASO-Gapmer), we could significantly prolong the therapeutic action of a single treatment up to 10 days. This study establishes FXYD2-LASO-Gapmer administration as a promising and efficient therapeutic strategy for long-lasting relief of chronic pain conditions in human patients.

## Introduction

A pain that lasts more than 3 months is considered as chronic (1, 2) and has lost the protective character associated with acute pain. Chronic pain, which affects 18% of the general population of developed countries and more than 1 billion people worldwide (3), can result from nerve damage (neuropathic pain) or inflammation (inflammatory pain), and its control is a major unresolved medical and socioeconomic problem (4). Current treatments present insufficient efficacy and often cause serious side effects (5–9). The care of patients suffering from chronic pain thus remains difficult, causing an important decrease in their quality of life and productivity (4, 10).

Chronic inflammatory pain and chronic neuropathic pain have distinct mechanisms and are treated differently, but they both engender highly disabling and persistent sensory abnormalities such as hyperalgesia and mechanical allodynia. Inflammatory pain results from the inflammatory process associated with tissue damage. Neuropathic pain results from a lesion or disease of the somatosensory system secondary to nerve trauma (accidental or surgical), viral infections, cancer chemotherapies, diabetes, or other autoimmune diseases. Approximately 7%–10% of the population suffers from neuropathic pain, and the incidence is increasing owing to the aging global population (11). There is thus an urgent need for developing novel approaches and discovering novel analgesic molecules to treat chronic pain. In addition, in some cases, inflammation can produce neuronal lesions that cause neuropathic pain, which in turn can trigger an inflammatory reaction and thus induce inflammatory pain. Therefore, another challenging parameter to take into account when developing a treatment is that chronic pain can be the consequence of a combination of inflammatory and neuropathic events.

We recently focused our attention on the *Fxyd2* gene, which encodes the γ subunit of the Na,K-ATPase pump. This gene exhibits a highly restricted expression profile throughout the body (12). It is notably found at high levels in kidneys (12, 13), and it is also present in specific subsets of somatosensory neurons of the dorsal root ganglia (DRG) in rodents and humans (14–16). Functional studies on constitutive *Fxyd2*-mutant mice using models of neuropathic and inflammatory pain have revealed that *Fxyd2* plays a proactive role in the chronification of pain (15, 16). Furthermore, we have demonstrated that acute inhibition of *Fxyd2* expression in DRG neurons of neuropathic rats in which a chronic pain state has been experimentally induced by nerve injury triggers a reduction of mechanical allodynia (16). This thus opens the perspective that the development of a therapeutic approach affecting the expression level and/or function of FXYD2 may represent a new strategy to alleviate chronic pain symptoms in human patients.

The use of antisense oligonucleotides (ASOs) and siRNA technology has emerged as an efficient approach to decrease the expression of a given protein or mRNA. Interestingly, oligonucleotide therapeutics are being increasingly developed in pharmaceutical research and successfully used in the clinic. As of 2020, more than 70 ASOs were in clinical trials (phase II or III) for cancer therapy, rheumatoid arthritis, asthma, and Duchenne muscular dystrophy (dystrophin exon 23), for example, and 9 are already on the market (including volanesorsen for management of hypertriglyceridemia and givosiran for treatment of acute hepatic porphyria). In particular, oligonucleotide therapies are attracting increasing attention for treating disorders affecting the nervous system (17), with 10 in clinical trials and 5 drugs already on the market (18): eteplirsen and golodirsen for treatment of some forms of Duchenne myopathy, inotersen and patisiran for treatment of familial amyloid polyneuropathy and cardiomyopathy (19), and nusinersen (Spinraza) for treatment of spinal amyotrophy (20).

The aim of this study was to develop a new therapeutic approach for treating chronic pain in human patients based on the use of antisense molecules targeting the *FXYD2* mRNA. To do so, we identified, designed, and validated a series of ASOs directed against the human *FXYD2* mRNA with high knockdown potential in HEK cells. Among the candidate target sequences, we identified a unique stretch of 20 nucleotides strikingly conserved in rats and humans, which was subsequently selected to set up a therapeutic protocol in vivo. We first established that intrathecal injections of an in-house-synthesized lipid-modified ASO targeting this sequence (FXYD2-LASO) led to efficient reduction of FXYD2 protein levels in rat DRG neurons, without the use of transfection reagent. We demonstrated that this FXYD2-LASO dramatically attenuated pain behaviors in rat models of neuropathic and inflammatory pain, without obvious side effects. We also report that injections of custom-made lipid-modified FXYD2-Accell siRNA directed against the same evolutionarily conserved region had similar beneficial impacts on pain symptoms. Finally, we synthesized a stabilized version of FXYD2-LASO using the 2′-*O*-methoxyethyl chemical modification (2′-MOE-FXYD2-LASO-Gapmer) and showed that this therapeutic molecule, intrathecally or intravenously injected, had potent and long-lasting analgesic effects in neuropathic and inflammatory pain. Taken together, these findings open up new perspectives for treating chronic pain conditions in human patients.

## Results

### Characterization of ASOs targeting the human FXYD2 mRNA.

**Figure 1:**
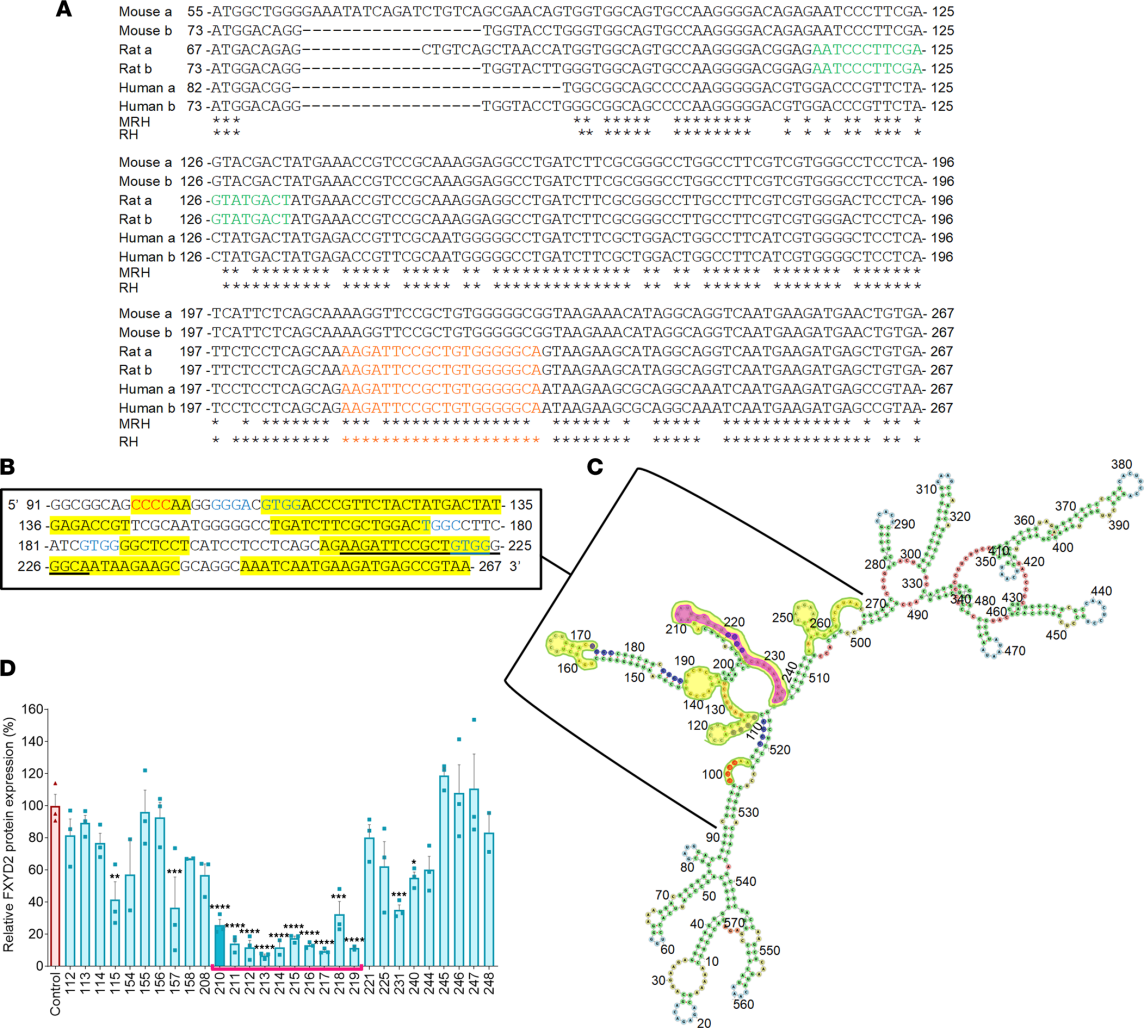
Identification of ASOs directed against human *FXYD2* mRNA. (**A**) Alignment of 2 variants (a and b) of the mouse (M), rat (R), and human (H) *FXYD2* gene coding sequences. The 20-mer and 100% conserved rat-human sequence, used to generate FXYD2-ASO that served as a model to test its efficacy in vivo using rat models, is outlined in orange. The sequence targeted by custom-made ON-TARGETplus siRNA in our previous study is shown in green. Interspecies homology is indicated by an asterisk. (**B**) mRNA sites containing multiloops or hairpin loops are identified in yellow, positive motifs in blue, and negative motifs in red in the common sequence encoding the 2 variants (a and b) of the human *FXYD2* mRNA. The 100% conserved rat-human sequence of FXYD2-LASO that we used for in vivo tests in the rat model is underlined. (**C**) The secondary structure of human *FXYD2* mRNA as predicted by the RNAfold program. Numbers represent the bases of the mRNA in the RefSeq annotation NM_001680. The region outlined in pink illustrates the part of the mRNA identified as susceptible to antisense inhibition in HEK293M cells. (**D**) Quantification of FXYD2 protein levels by Western blot of extracts of HEK293M cells after transfection by a series of ASOs directed against human *FXYD2* mRNA. The levels of FXYD2 protein were normalized to actin protein. Data are represented as means ± SEM, *n* = 3 replicates. One-way ANOVA and post hoc Bonferroni’s test. *P < 0.05; **P < 0.01; ***P < 0.001; ****P < 0.0001.

We previously showed that knockdown of *Fxyd2* in rat DRG by intrathecal injection of custom-made ON-TARGETplus siRNA (Horizon Discovery Ltd.) (green sequence in Figure 1A) using transfection reagents can reverse established neuropathic pain behavior. However, the target sequence of this siRNA was rat specific and can thus not be transposed to humans. Moreover, although siRNAs are being widely used, most current antisense therapeutic strategies are based on antisense oligonucleotides (ASOs) (21). Therefore, in order to develop an ASO-based therapy adaptable to humans to treat chronic pain, we first aimed at identifying relevant regions in the human *FXYD2* mRNA that could be efficiently targeted by ASOs. To be effective, ASOs must meet several criteria. Their ideal size is 20 nucleotides, their GC composition should not exceed 60%, and their activity is correlated with the presence of so-called positive motifs and absence of negative motifs (22). Positive motifs are GCCA (UGGC in mRNA), CCAC (GUGG mRNA), ACTC (GAGU mRNA), TCCC (GGGA mRNA), and CTCT (AGAG mRNA), and several were identified in the 2 variants (a and b) of *FXYD2* (blue sequences in Figure 1B). Negative motifs are GGGG (CCCC mRNA), ACTG (CAGU mRNA), TAA (UUA mRNA), AAA (UUU mRNA), and CCGG (CCGG mRNA), one of which was found in the *FXYD2* sequence (in red in Figure 1B). Moreover, secondary mRNA structures forming multi-loops or hairpin loops have also been reported to be most susceptible to efficient antisense targeting (23). Analysis of the secondary structure of the human *FXYD2a* mRNA (NCBI reference sequence NM_001680) determined by RNAfold software (http://rna.tbi.univie.ac.at/cgi-bin/RNAWebSuite/RNAfold.cgi) revealed the presence of such favorable structures in the common sequence encoding the human *FXYD2a* and *b* variants (yellow regions in Figure 1, B and C). Taking all these criteria into account, we designed a series of 29 overlapping ASOs (Supplemental Figure 1A; supplemental material available online with this article; https://doi.org/10.1172/jci.insight.161246DS1) covering bases 112–267 of the human *FXYD2* mRNA that were synthesized using the classic phosphorothioate chemical modifications to increase half-life, cellular uptake, and specificity (24). The functional knockdown property of each ASO was tested in vitro by transfection into human HEK293M cells — which endogenously express high levels of FXYD2 — and quantification of FXYD2 protein levels by Western blot. The region encompassing bases 210–238 (targeted by ASO210–219) was found to be particularly favorable for antisense inhibition (~67% to 93% inhibition) (pink region in Figure 1C; pink brackets in Figure 1D). Remarkably, we found that within this region, ASO210 targets a sequence in the *FXYD2* mRNA that is identical in rats and humans and is also common to both *FXYD2a* and *b* variants. Importantly, this target sequence (in orange in Figure 1A and underlined in Figure 1B) meets several important criteria: its composition in GC is 60%, it contains 1 positive motif, it is devoid of negative motifs, and its secondary structure includes several loops.

We next tested whether shorter sequences of 15 or 17 nucleotides of some of the 20-mer ASO candidates may be similarly efficient in affecting FXYD2 expression in HEK cells. We particularly focused on the evolutionarily conserved ASO210, which reduces FXYD2 expression level by about 74%, and ASO217, which shows the higher score by triggering a 90% FXYD2 protein reduction (Figure 1D). Comparative analyses of the 15-, 17-, and 20-mer versions of ASO210 or ASO217 revealed that for both of them, maximum inhibition was achieved with lengths of 20 nucleotides (Supplemental Figure 1B). Taken together, these in vitro studies allowed us to identify a region within the coding sequence of the human *FXYD2* mRNA particularly favorable for efficient gene knockdown using 20-mer ASOs, which strikingly contains a unique stretch of 20 nucleotides 100% conserved between rat and human.

### Efficient knockdown of FXYD2 by lipid-modified form of ASO210 without the use of transfection reagents.

**Figure 2:**
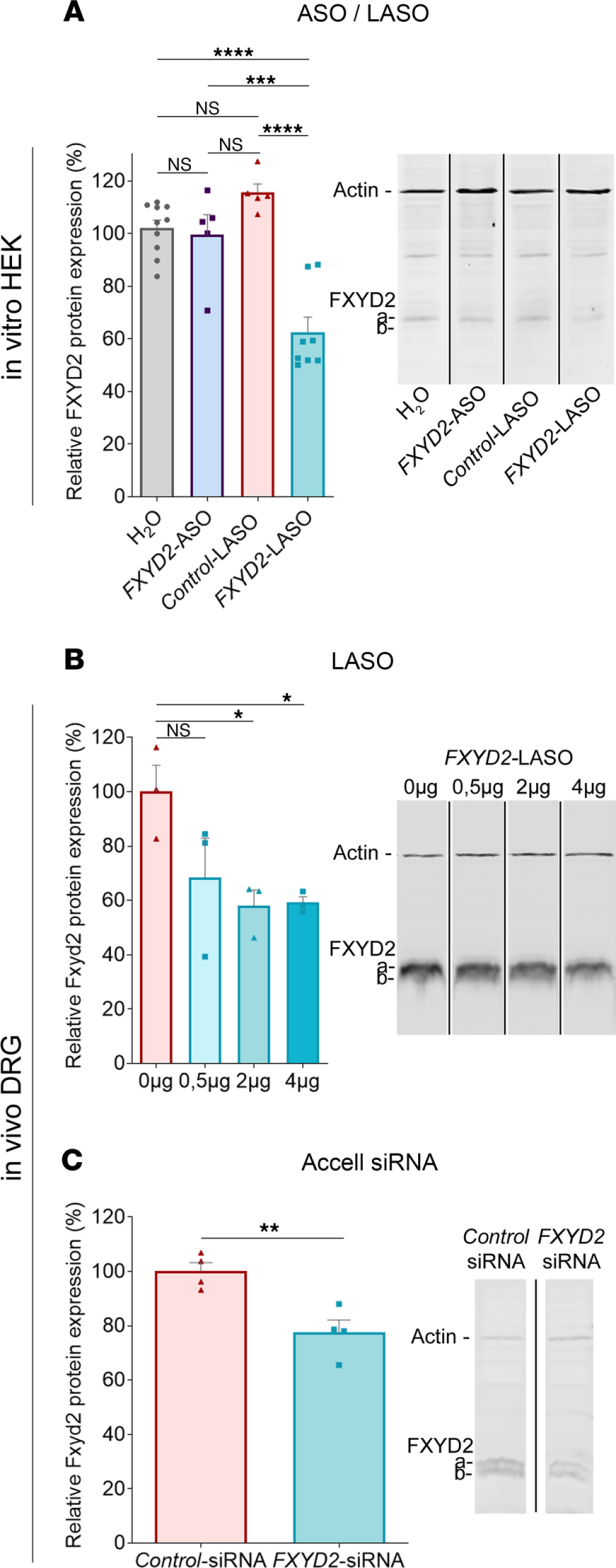
FXYD2 expression can be inhibited in DRG by intrathecal injection of ASOs without the use of toxic transfection reagents. (**A**) Quantification of FXYD2 protein levels by Western blot of extracts of HEK293M cells after transfection by FXYD2-ASO or FXYD2-LASO without transfection reagents. (**B** and **C**) Increasing amounts (0, 0.5, 2, and 4 μg) of FXYD2-LASO (**B**) or 2 μg of FXYD2-Accell siRNA (**C**) were intrathecally injected daily over 14 days (*n* = 3). Lumbar DRG (L4 and L5) tissues were dissected and quantified for FXYD2 protein levels. Western blot using an FXYD2 antibody shows the presence of the FXYD2 isoforms a and b. The levels of FXYD2 protein were normalized to actin protein. Lanes were transferred onto the same membrane but were noncontiguous. Data are represented as means ± SEM of data from 5–10 replicates (**A**), 3–4 animals (**B** and **C**). One-way ANOVA and post hoc Bonferroni’s test (**A** and **B**), 2-tailed unpaired Student’s *t* test (**C**). *P < 0.05; **P < 0.01; ***P < 0.001; ****P < 0.0001.

Because of its evolutionary conservation and its high inhibition efficiency in vitro, we selected the ASO210 sequence for functional analyses in vivo in rats. First, to avoid the potential deleterious effects of transfection reagents on cell survival, we tested an alternative cell delivery strategy based on the use of lipid modifications described by Pokholenko et al. (25). A lipid-modified form of ASO210, hereafter called FXYD2-LASO, was thus synthesized and incubated with HEK293M cells without transfection reagents, and its impact on endogenous FXYD2 expression was evaluated. We found that while FXYD2-ASO (without lipid modification or transfection reagent) and control-LASO (lipid-modified “nontargeting” ASO) had virtually no effect, FXYD2-LASO significantly downregulated FXYD2 protein expression levels by about 37% (Figure 2A). This prompted us to assess the efficiency of FXYD2-LASO in vivo in rat DRG neurons. To test this, we carried out a dose-response analysis to determine the minimum amount of FXYD2-LASO that effectively decreases FXYD2 protein levels in rat DRG. We performed daily intrathecal injections at lumbar levels of either 0 μg, 0.5 μg, 2 μg, or 4 μg of FXYD2-LASO for 14 days, and the fourth and fifth lumbar DRGs (L4 and L5) were subsequently dissected out and analyzed by Western blot. By doing so, we established that injections of FXYD2-LASO were indeed able to downregulate FXYD2 expression in vivo and that maximal knockdown (~42%) was achieved with 2 μg of FXYD2-LASO as no further decrease was observed using higher amounts (Figure 2B). In parallel, we also tested the efficacy of using siRNAs as an alternative method. A custom-made 20-mer siRNA directed against the same 100% conserved region of the rat and human *FXYD2* genes was synthesized using the Accell technology (Horizon Discovery Ltd.) reported to also facilitate cell penetration without transfection reagent. In this experimental paradigm, we injected daily 2 μg of FXYD2-Accell siRNA or “nontargeting”-Accell siRNA as control for 14 days. Again, we observed that FXYD2-Accell siRNA was specifically able to downregulate the level of FXYD2 protein in L4 and L5 DRG by about 23% (Figure 2C).

Taken together, these results established that lipid-modified FXYD2-LASO and FXYD2-Accell siRNA represent potent molecules able to efficiently downregulate FXYD2 expression in vitro and in vivo without the use of transfection reagent.

### FXYD2-LASO or FXYD2-Accell siRNA treatments alleviate mechanical hypersensitivity in a neuropathic chronic pain model.

**Figure 3:**
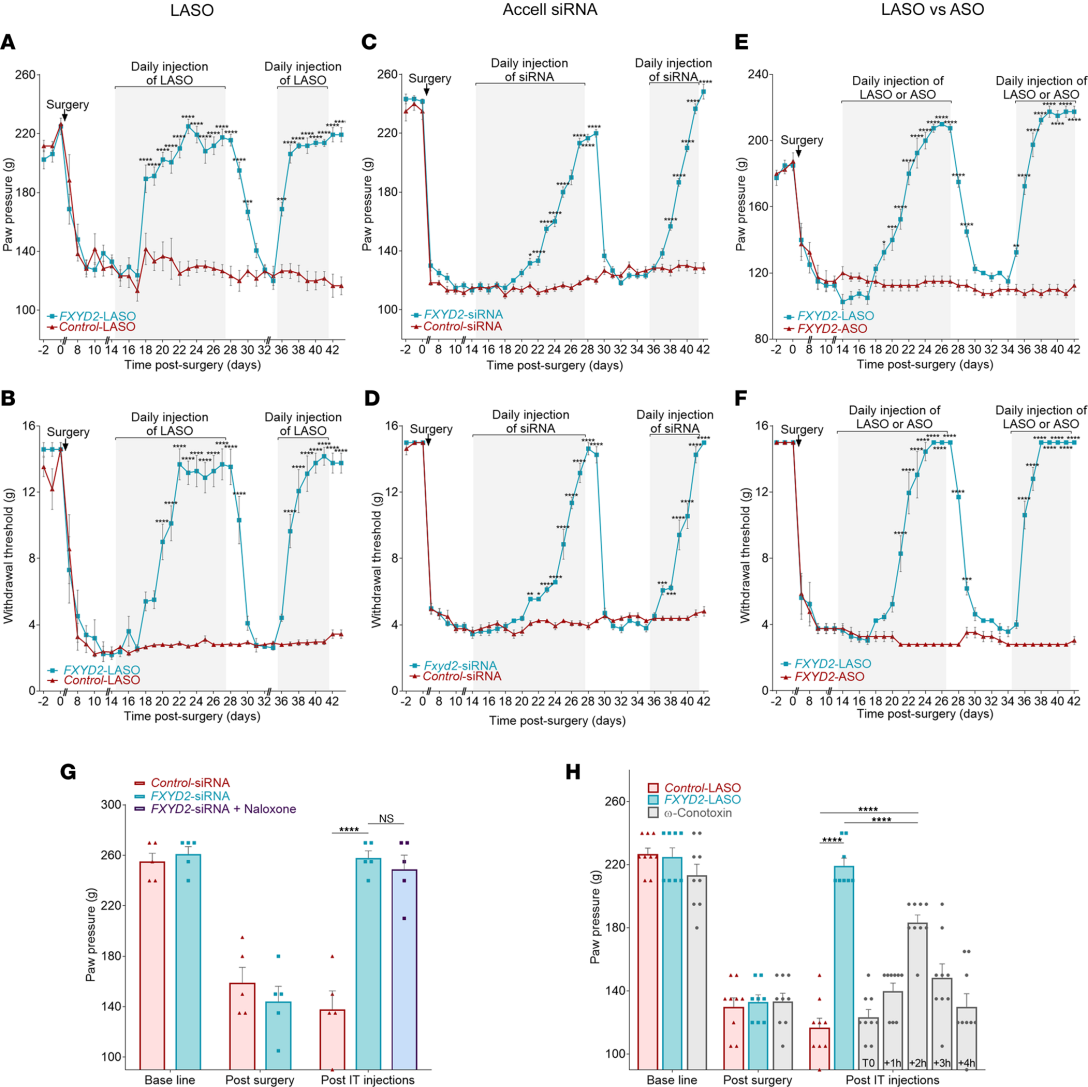
FXYD2 inhibition by intrathecal injection of FXYD2-LASO or FXYD2-Accell siRNA reduces mechanical hypersensitivity in the neuropathic pain model (SNL); this treatment does not act by modulating the activity of the opioid receptor and is at least as effective as ω-conotoxin MVIIA for pain relief. (**A**–**F**) After induction of mechanical hypersensitivity by SNL, male rats were intrathecally injected daily for 14 days with FXYD2- or control-LASO (**A** and **B**), FXYD2- or control-Accell siRNA (**C** and **D**), and FXYD2-LASO or FXYD2-ASO (**E** and **F**). FXYD2-LASO and FXYD2-Accell siRNA, but not control-LASO, control-Accell siRNA, and FXYD2-ASO, reduced mechanical hypersensitivity in Randall-Selitto (**A**, **C**, and **E**) and von Frey (**B**, **D**, and **F**) tests. Gradual complete alleviation of mechanical hypersensitivity in both tests was reversed by interruption of FXYD2-LASO or FXYD2-Accell siRNA injections, which caused a return of hypersensitivity to mechanical stimuli within 2 days. The resumption of the injections during 7 days restored the analgesic effect in 4–6 days. (**G** and **H**) After SNL surgery, cohorts of rats displaying neuropathic pain symptoms were intrathecally injected daily for 14 days with FXYD2- or control-Accell siRNA (**G**) or FXYD2- or control-LASO (**H**). When complete attenuation of pain behavior was obtained in the cohort of rats treated with FXYD2-Accell siRNA, a single intraplantar injection of naloxone, a morphine receptor antagonist, failed to induce mechanical hypersensitivity and a pain reaction 5 minutes after the injection. When complete attenuation of pain behavior was obtained in the cohort of FXYD2-LASO–treated rats, a separate cohort of neuropathic rats was treated with a single intrathecal injection of ω-conotoxin MVIIA. Conotoxin treatment triggers an analgesic effect, which was lower than FXYD2-LASO and which lasted only 2 hours. Means ± SEM of data from 9 (**A**–**D**), 5–6 (**E**–**G**), and 8–9 (**H**) animals. Two-way ANOVA and post hoc Bonferroni’s test. *P < 0.05; **P < 0.01; ***P < 0.001; ****P < 0.0001.

We then tested the potential analgesic effect of intrathecal injections of FXYD2-LASO (Figure 3, A and B) and FXYD2-Accell siRNA (Figure 3, C and D) in vivo using the spinal nerve ligation (SNL) model of peripheral neuropathic chronic pain in rats (26). As expected, 8 days after the SNL surgery and before any treatment, SNL rats all displayed persistent hypersensitivity to mechanical stimuli as evidenced by increased responses to paw pressure (Randall-Selitto test; Figure 3, A and C) and lowered withdrawal thresholds to von Frey filaments (Figure 3, B and D) on their ipsilateral paw compared with baseline levels or control sham rats (Supplemental Figure 2, A–D). Strikingly, daily intrathecal injections of 2 μg of either FXYD2-LASO or FXYD2-Accell siRNA from day 14 post-surgery, after pain chronification, caused a gradual reversal of pain behaviors, and the responses returned to baseline levels after 10 or 14 injections, respectively (Figure 3, A–D). Moreover, we determined that complete attenuation of pain behavior was maintained as long as FXYD2-LASO or FXYD2-Accell siRNA treatments continued. Indeed, interruption of the injections caused a return of mechanical hypersensitivity within 2 days. Nevertheless, resumption of FXYD2-LASO or FXYD2-Accell siRNA treatments restored the analgesic effect after only 4 or 6 injections, respectively, which was much faster than for the initial phase of injections (respectively 10 and 14 days; see above and Figure 3, A–D). Importantly, we showed that injections of 2 μg of control-LASO or control-Accell siRNA in neuropathic rats had no attenuating effect on their pain behavior (Figure 3, A–D; see also Supplemental Figure 2, A–B′). In addition, control- and FXYD2-LASO or control- and FXYD2-Accell siRNA also had no effect on the sensitivity of control sham rats (Supplemental Figure 2, A–B′). We also determined that groups of male (Figure 3) and female (Supplemental Figure 3, A and A′) rats exhibited similar sensitivity to mechanical stimulations in all experimental paradigms. In particular, injections of FXYD2-LASO in neuropathic females also efficiently alleviated their pain symptoms, thus revealing no sex discrepancy of the FXYD2-LASO–based analgesic treatment (Supplemental Figure 3, A and A′). Finally, we further assessed the importance of the lipid modification by directly comparing the responses of neuropathic rats to intrathecal injections of non-modified FXYD2-ASO and lipid-modified FXYD2-LASO (Figure 3, E and F). After SNL surgery and establishment of long-term neuropathic pain behavior, cohorts of rats were treated by daily injections of 2 μg of either FXYD2-ASO or FXYD2-LASO. Mechanical sensitivity testing on these animals showed that, in contrast to FXYD2-LASO, FXYD2-ASO injections were inefficient for attenuating pain behaviors. This thus demonstrates the absolute necessity of the lipid modification to ensure efficiency of the treatment.

We next tested whether intrathecal injections of FXYD2-LASO and FXYD2-Accell siRNA had only a local action on *Fxyd2* expression within the DRG or a more global impact throughout the organism. To do so, at the end of each experiment, we systematically dissected out L4 and L5 DRG as well as the kidneys, where FXYD2 is highly expressed (12, 13), and we quantified FXYD2 protein levels by Western blot. Results on the DRG always revealed a significant inhibition of FXYD2 protein levels by about 36% to 44% (Supplemental Figure 4, A–C). In contrast, we observed no effect in kidneys (Supplemental Figure 4, A′, B′, and C′). This thus supports that intrathecal injections of FXYD2-LASO or FXYD2-Accell siRNA act locally on somatosensory neurons but do not have a systemic effect.

Peripheral nerve injury is known to trigger the down- or upregulation of a battery of genes in the DRG. The analgesic effect of FXYD2 knockdown prompted us to assess whether the FXYD2-LASO treatment could impinge on these molecular changes by using the NanoString nCounter gene expression quantification system. We extracted total RNA from ipsilateral L5 DRG from 3 animals 6 weeks after surgery and treatments, from 3 different animal cohorts: control-LASO–treated sham animals, control-LASO–treated SNL animals, and FXYD2-LASO–treated SNL animals that had recovered normal sensitivity. We focused our analysis on 43 genes listed in Supplemental Figure 4D, which were selected based on previous transcriptomic studies and literature (27, 28). These notably include the upregulated genes *ATF3*, *Csf1*, and *Gadd45a* and the downregulated genes *MrgPrd*, *Kcns1*, and *Hapln4*. Comparative analysis between control-LASO–treated sham animals and control-LASO–treated SNL animals validated the approach since the list of up- and downregulated genes after SNL was fully consistent with previous studies (27, 28). Remarkably, the overall gene expression profile of the 43 selected genes in the DRG of FXYD2-LASO–treated SNL rats was largely similar compared with control-LASO–treated SNL rats (Supplemental Figure 4D). This result suggests that the mechanism by which FXYD2-LASO alleviates mechanical hypersensitivity in neuropathic pain models does not involve major changes in gene expression in DRG neurons.

Next, we aimed at determining whether the FXYD2-LASO treatment may cause global side effects. To test this, we evaluated the general health and behavior of sham animals treated 14 days with daily intrathecal injections of 2 μg of control- (*n* = 6) or FXYD2-LASO (*n* = 6). Daily observation of the different animal cohorts during the whole period of treatment did not reveal any behavior of restlessness or prostration, spontaneous vocalization, weight loss (Supplemental Figure 4E), breathing difficulties, or abnormal appearance of the coat. In addition, using the open field test, we established that the velocity and duration of the time spent at the center or at the periphery of the open field arena did not change between groups of rats (Supplemental Figure 4, F–G′). These results thus suggest that FXYD2 inhibition achieved with FXYD2-LASO has virtually no effect on general locomotor activity and anxiety. All together, these data establish that knockdown of FXYD2 expression in the DRG induced by intrathecal injections of lipid-modified ASOs or Accell siRNA constitutes an efficient and safe therapeutic approach to treat peripheral neuropathic chronic pain.

### The pain-relieving effect of FXYD2-LASO treatment is independent of opioid signaling system and is as potent as that of ω-conotoxin MVIIA.

The endogenous opioid system plays a central role in acute pain, and exogenous opioid molecules, including morphine, represent one of the most efficient analgesic drug families to date (29). This prompted us to investigate whether the analgesic effects induced by the FXYD2 antisense treatments involved the opioid system. To test this, we assessed whether subcutaneous injections of naloxone — an opioid receptor antagonist — into FXYD2-Accell siRNA–treated SNL rats that had regained normal sensitivity re-induced mechanical allodynia (30). However, we found that in this experimental paradigm, naloxone did not block the analgesic effect of the FXYD2-Accell siRNA treatment (Figure 3G). This result supports that the pain relief induced by FXYD2 knockdown in neuropathic rats does not involve modulations of opiate receptor activity and is therefore opioid independent.

We next compared the impact of FXYD2 antisense molecules versus ω-conotoxin MVIIA, the current gold standard intrathecally administered medication commercialized under the name Prialt. This molecule is a calcium channel inhibitor used in certain cases of intractable pain in humans, which has been reported to have a quick and transient analgesic effect (31). Because of obvious temporal divergences of both treatments (31) (Figure 3, A and B, and below), we particularly focused on the maximum analgesic effect triggered by intrathecal injections of FXYD2-LASO and ω-conotoxin in neuropathic rats. In this experiment, we analyzed 3 cohorts of SNL rats in parallel. A first cohort was injected daily with control-LASO and served as control. A second cohort was treated daily with FXYD2-LASO until their mechanical sensitivity returned to normal levels and reached a plateau at day 14. A that point, the third cohort was injected intrathecally with 100 pmol of ω-conotoxin, and a time-course analysis of their mechanical sensitivity was performed over a 4-hour period. In line with previous observations, the analgesic effects of ω-conotoxin started 1 hour after injections, peaked at 2 hours, and eventually ceased after 4 hours (Figure 3H). Interestingly, the score of FXYD2-LASO–treated neuropathic animals using the Randall-Selitto test at day 14 was significantly higher than the highest score of ω-conotoxin registered at 2 hours. Thus, while ω-conotoxin MVIIA has a quicker but partial and short-lived effect, FXYD2-LASO appears to be more efficient with an extended duration, but with a longer onset time.

Together these results show that FXYD2 antisense treatment for neuropathic pain is independent of the opioid system and is as efficient, if not more, than the current gold standard ω-conotoxin MVIIA, though with distinct temporal dynamics.

### FXYD2-LASO or -Accell siRNA treatment alleviates mechanical hypersensitivity in an inflammatory pain model.

**Figure 4:**
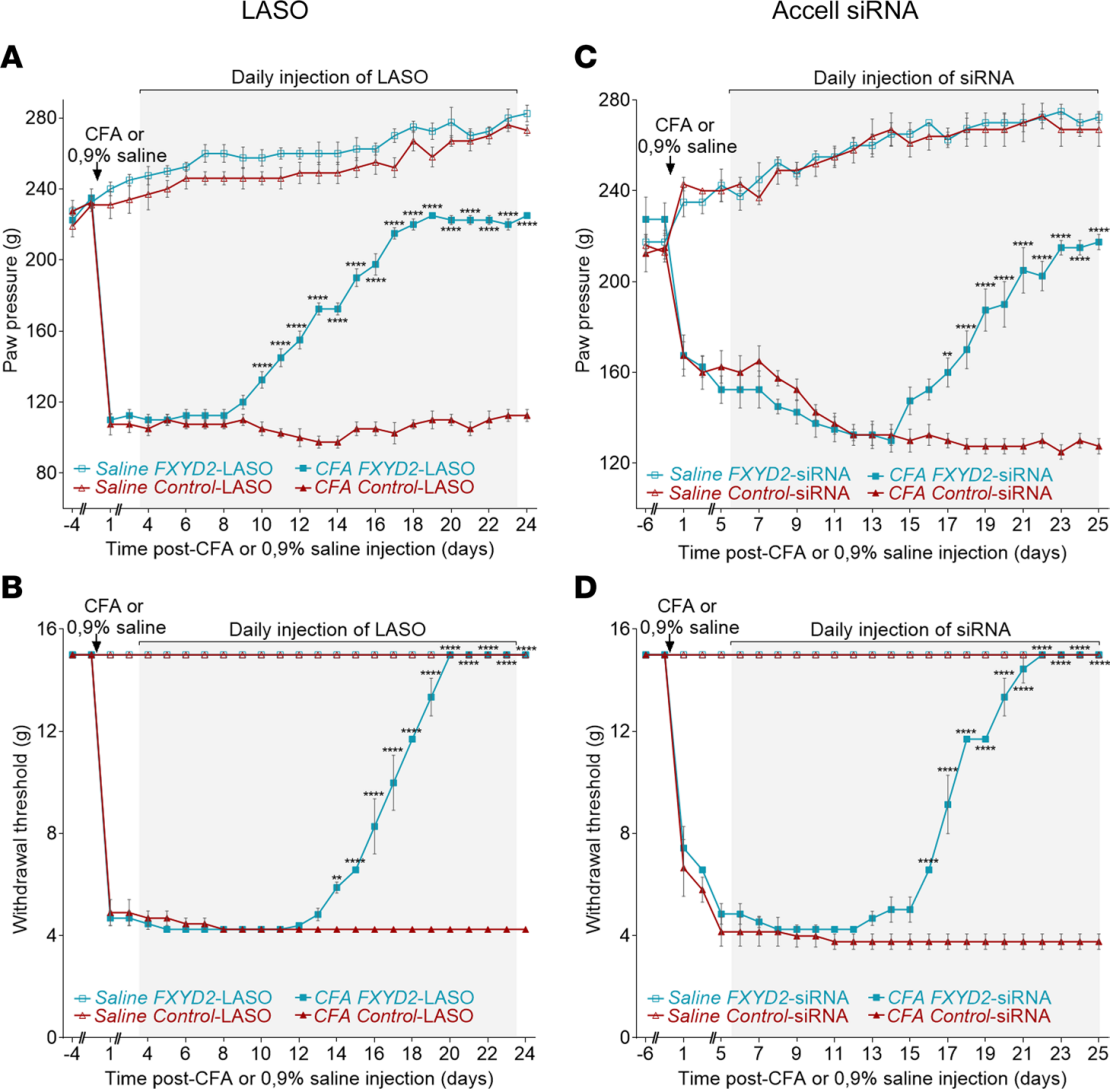
FXYD2-LASO or FXYD2-Accell siRNA treatment alleviates mechanical hypersensitivity in an inflammatory pain model. Mechanical hypersensitivity was induced by intraplantar injection of CFA. Daily injections of FXYD2-LASO (**A** and **B**) or FXYD2-Accell siRNA (**C** and **D**) during 21 days, but not control-LASO or control-Accell siRNA, alleviated pain-related behavior (**A** and **C**, Randall-Selitto paw pressure test; **B** and **D**, von Frey test). FXYD2- and control-LASO or FXYD2- and control-Accell siRNA had no effect on saline rats’ behavior. Means ± SEM of data from 6 animals. Two-way ANOVA and post hoc Bonferroni’s test. **P < 0.01; ****P < 0.0001.

We next tested whether FXYD2 inhibition by FXYD2-LASO and FXYD2-Accell siRNA could also be effective in other chronic pain models, where the underlying mechanisms are known to be different from those pertaining in nerve injury–induced pain. We employed the commonly used inflammatory pain model of intraplantar injection of complete Freund’s adjuvant (CFA), which causes a long-lasting mechanical hypersensitivity. Again, mechanical sensitivity was tested using the Randall-Selitto test (Figure 4, A and C) and von Frey filaments (Figure 4, B and D). As expected, injection of CFA caused a rapid mechanical hypersensitivity within 2 days that was maintained over the time course of the experiment in control animals treated with control-LASO or control-Accell siRNA. In contrast, daily intrathecal injections of 2 μg of FXYD2-LASO (Figure 4, A and B) or FXYD2-Accell siRNA (Figure 4, C and D) attenuated the pain behavior, albeit in a slightly longer time frame compared with neuropathic pain models. Indeed, the maximum analgesic effect was achieved after 16–18 days in the inflammatory pain model compared with 10–14 days in the neuropathic pain model (see above). Note that, again, control- and FXYD2-Accell siRNA or control- and FXYD2-LASO had no effect on sham rats (Figure 4, A–D). Moreover, no sex difference was observed using the CFA model of inflammatory pain, males (Figure 4) and females (Supplemental Figure 3, B and B′) exhibiting similar responses in all tests and conditions. These results thus show that the use of FXYD2-LASO or FXYD2-Accell siRNA as treatment can be beneficial, not only for neuropathic pain, but also for inflammation-induced chronic pain.

### 2′-MOE–modified FXYD2-LASO-Gapmer provides long-lasting pain relief in neuropathic and inflammatory pain models.

**Figure 5:**
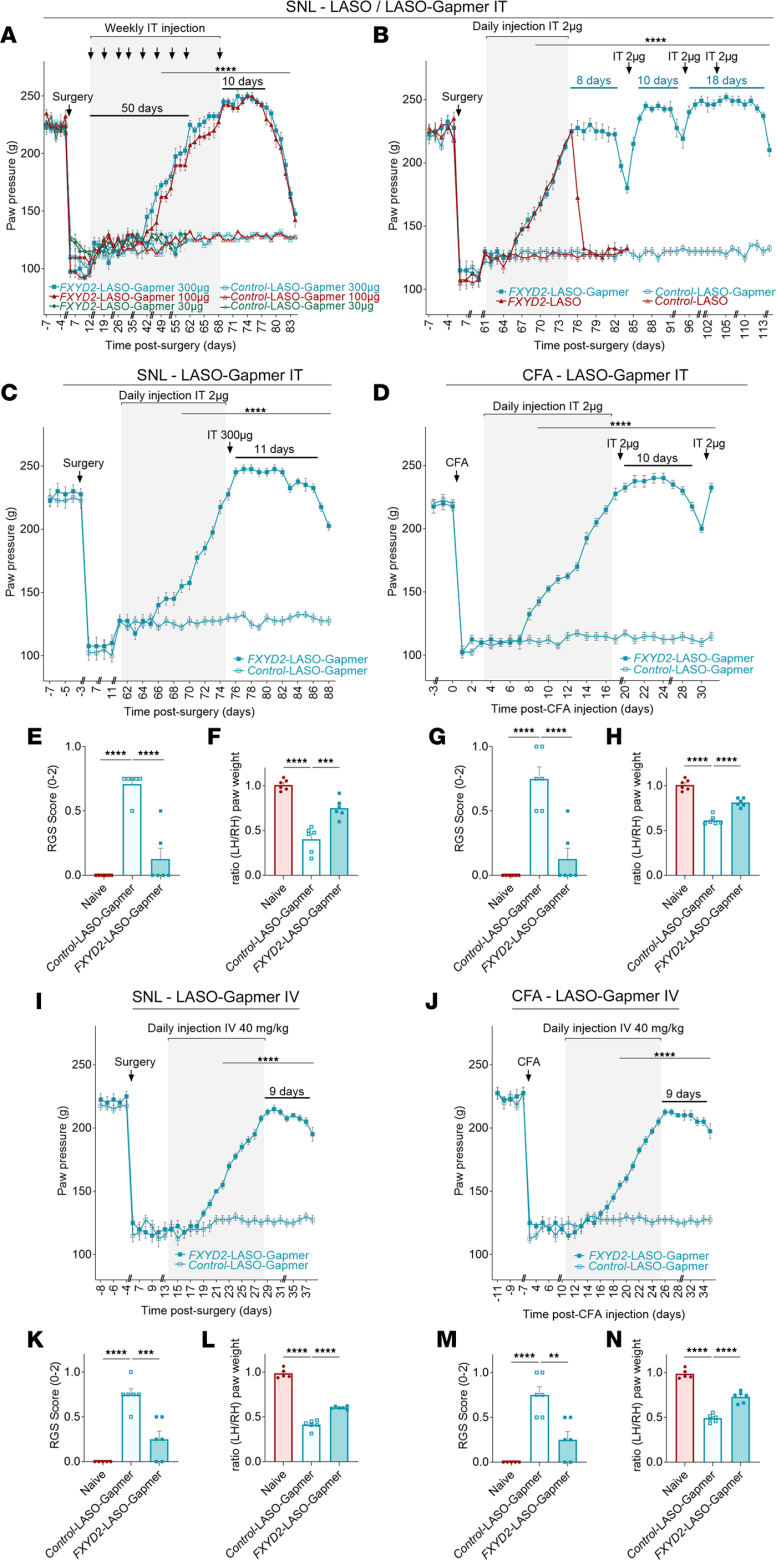
2′-MOE–modified FXYD2-LASO-Gapmer provides long-lasting pain relief in neuropathic pain and inflammatory pain models. (**A**–**C**) After induction of mechanical hypersensitivity by SNL, animals were intrathecally injected weekly — until full recovery — for 56 days with 30, 100, or 300 μg of FXYD2- or control-LASO-Gapmer (**A**); daily for 14 days with 2 μg of FXYD2- or control-LASO or FXYD2- or control-LASO-Gapmer — until full recovery — followed by an injection every 8–11 days of 2 μg of FXYD2- or control-LASO-Gapmer (**B**); or daily for 14 days with 2 μg followed by a 300 μg single injection of FXYD2- or control-LASO-Gapmer (**C**). (**D**) Mechanical hypersensitivity was induced by intraplantar injection of CFA, and animals were intrathecally injected daily for 15 days with 2 μg of FXYD2- or control-LASO-Gapmer and reinjected with a 2 μg single injection of FXYD2- or control-LASO-Gapmer when a return of hypersensitivity to mechanical stimuli was observed. (**E**–**H**) To test spontaneous pain, the Rat Grimace Scale (RGS) (**E** and **G**) and Static Weight Bearing (SWB) test (**F** and **H**) were used when complete attenuation of pain behavior was reached and assessed by Randall-Selitto evoked behavioral test in cohort of SNL (**E** and **F**) or CFA (**G** and **H**) rats intrathecally injected daily for 14 days with 2 μg of FXYD2-LASO-Gapmer. (**I**–**N**) After induction of mechanical hypersensitivity by SNL (**I**, **K**, and **L**) or CFA (**J**, **M**, and **N**), animals were intravenously injected daily for 16 days with 40 mg/kg of FXYD2- or control-LASO-Gapmer (**I** and **J**). After the last injection (day 28 in **I**, day 25 in **J**), RGS (**K** and **M**) and SWB test (**L** and **N**) were performed to evaluate spontaneous pain. Mechanical hypersensitivity was evaluated with the Randall-Selitto test (**A**–**D**, **I**, and **J**). Means ± SEM of data from 6 animals (**A**–**N**). Two-way (**A**–**D**, **I**, and **J**) and 1-way (**E**–**H** and **K**–**N**) ANOVA and post hoc Bonferroni’s test. **P < 0.01; ***P < 0.001; ****P < 0.0001.

Although highly efficient, the treatment based on FXYD2-LASO requires daily intrathecal injections over long periods, which may represent an obstacle for its routine application in clinical practice. In order to circumvent this potential problem, we tested whether 2′-*O*-2-methoxyethyl chemical modifications, known to significantly increase the metabolic stability and binding affinity of ASOs to their target mRNA sequence (32), may be advantageous. We thus synthesized 2′-*O*-2-methoxyethyl–modified FXYD2-LASO (hereafter referred to as FXYD2-LASO-Gapmer) and evaluated its efficiency in rat models of chronic pain. First, we assessed the in vivo effects of weekly intrathecal injections FXYD2-LASO-Gapmer at 3 different doses (30 μg, 100 μg, or 300 μg) in neuropathic rats using the SNL model. We found that while 30 μg doses were ineffective, 8 injections of either 100 μg or 300 μg over 50 days were needed to achieve complete alleviation of pain (Figure 5A), which was much longer than the 10–14 days required with daily injections of 2 μg of FXYD2-LASO (Figure 3, A and B, and Figure 4, A and B). Remarkably however, after interruption of the FXYD2-LASO-Gapmer treatment, the analgesic effects lasted for 10 days (Figure 5A), while they lasted for only 2 days after the last dose of FXYD2-LASO (Figure 3, A and B). This shows that while weekly injections of even high doses of FXYD2-LASO-Gapmer seem not beneficial compared with daily injections of 2 μg of FXYD2-LASO during the primary phase of recovery, they subsequently greatly extend the duration of the analgesic effects.

These observations prompted us to set up a hybrid protocol consisting of a primary phase of daily intrathecal injections of FXYD2-LASO-Gapmer until recovery was achieved, followed by a second phase of more spaced injections to maintain the analgesic effects over time. In this experimental paradigm, and based on our data with FXYD2-LASO, we envisaged injections of 2 μg of the molecules. We first compared the impact of daily intrathecal injections of 2 μg of either FXYD2-LASO or FXYD2-LASO-Gapmer. We found that both molecules were equally efficient in achieving complete attenuation of pain behavior within the same time frame of approximatively 13 days (Figure 5B). As shown before (Figure 3, A and B), pain relief lasted for only 1 or 2 days after interruption of the FXYD2-LASO injections. In contrast, analgesia was maintained 8–10 days after the last dose of FXYD2-LASO-Gapmer (Figure 5B). We also determined that higher doses of 300 μg did not significantly extend the duration of the analgesic effect between 2 injections (Figure 5C) during the second phase of the treatment. Next, we also tested this therapeutic protocol on CFA-induced inflammatory pain models. Again, we observed that complete attenuation of pain behavior was obtained by daily injections of 2 μg of FXYD2-LASO-Gapmer after 14 days and this analgesic effect was subsequently maintained by a single intrathecal injection every 10 days (Figure 5D).

Together these results show that the use of 2′-*O*-2-methoxyethyl chemical modifications to stabilize the FXYD2-LASO significantly enhances the efficacy of the treatment for both neuropathic and inflammatory chronic pain. Nevertheless, while these FXYD2-LASO-Gapmers significantly extend the period of the treatment’s efficiency in the second phase, they do not prevent the need for daily injections in the primary phase. This may reflect unusual stability of the FXYD2 protein and/or the slow action of the gapmer to initiate FXYD2 knockdown. To test this, we first determined the half-life of FXYD2 protein in HEK293M cells by using the cycloheximide chase method. To do so, 50 mg/mL cycloheximide was applied to halt protein synthesis by blocking the translation of mRNA. Cells were harvested at 0 hours (control) or 0.5, 1, 3, 6, 9, 12, 24, or 48 hours after cycloheximide treatment, and the amount of FXYD2 in control- and cycloheximide-treated cells (Supplemental Figure 5A) was assessed by Western blot. The relative amount of FXYD2 in cycloheximide-treated cells at each time point was compared with the control condition (0 hours). We found that the level of FXYD2 was reduced to 38.20% of the control after 12 hours of cycloheximide treatment (Supplemental Figure 5B). Based on the curve, FXYD2 levels were reduced to 50% at 11.4 hours after cycloheximide treatment, indicating that the half-life of FXYD2 is approximately 11 hours in HEK293M cells, which is in the median range (33). Next, we determined the time required to reach maximum reduction of FXYD2 levels in DRG of CFA rats injected daily with 2 μg of FXYD2-LASO-Gapmer. To do so, we evaluated the level of FXYD2 before (control) and after 3, 7, 10, and 14 intrathecal injections (IT), corresponding to the first phase of the treatment (Supplemental Figure 5C). Strikingly, we observed that only 3 daily intrathecal injections of FXYD2-LASO-Gapmer were sufficient to decrease the level of FXYD2 protein by 72% in L4 and L5 DRG (+3 IT; Supplemental Figure 5, D and E). However, recovery of normal mechanical sensitivity lagged behind the decrease in FXYD2 protein levels by several days (Supplemental Figure 5C). Together, these results suggest that the initial latency period for the treatment to be efficient cannot be simply explained by the long half-life of the FXYD2 protein or by the slow action of the therapeutic molecule to trigger FXYD2 knockdown, and likely reflects the establishment of transient compensatory pathological mechanisms.

The widespread and classical behavioral tests used in the experiments described above are designed to assess evoked pain. In rats treated with 14 daily intrathecal injections of FXYD2-LASO-Gapmer (2 μg) after induction of neuropathic (SNL; Figure 5, E and F) or inflammatory (CFA; Figure 5, G and H) pain, we also tested whether our treatment had a beneficial impact on spontaneous pain by monitoring changes in face grimace using the Rat Grimace Scale (RGS) (Figure 5, E and G), and by evaluating hind-paw weight distribution using the Static Weight Bearing (SWB) test (Figure 5, F and H). As expected, while naive groups exhibited no sign of spontaneous pain (score 0; Figure 5, E and G), the RGS scores of SNL and CFA groups treated with control-LASO-Gapmer were significantly increased (score of 0.75 for both groups; Figure 5, E and G). In contrast, the RGS scores of groups of SNL or CFA rats treated with FXYD2-LASO-Gapmer returned to significantly lower values (scores of 0.25 for both groups; Figure 5, E and G). This indicates that intrathecal injections of FXYD2-LASO-Gapmer also alleviate spontaneous pain. During the SWB test, the pressure exerted by each hind paw was recorded, and the weight balance was assessed by calculation of the ratio between the left and right hind paws. As expected, these ratios were close to 1.0 for naive rats, reflecting a balanced weight distribution on both hind paws (Figure 5, F and H). In contrast, in groups of SNL or CFA rats (with lesions on the left hind paw) treated with control-LASO-Gapmer, this ratio was significantly decreased (0.40 and 0.61, respectively; Figure 5, F and H). Strikingly, in SNL or CFA rats treated with FXYD2-LASO-Gapmer this ratio was significantly higher compared with control-LASO-Gapmer (0.75 and 0.79, respectively; Figure 5, F and H), though without reaching the levels of naive groups. These results indicated that intrathecal injections of FXYD2-LASO-Gapmer in rats with neuropathic and inflammatory pain significantly alleviated their spontaneous pain symptoms.

Although highly efficient, the treatment based on FXYD2-LASO requires daily intrathecal injections over long periods, which may represent an obstacle for its routine application in clinical practice. In order to circumvent this potential problem, we tested whether a less invasive intravenous route of administration of FXYD2-LASO-Gapmer could be advantageous. We thus assessed the in vivo effects of daily intravenous injections of FXYD2-LASO-Gapmer at 40 mg/kg in SNL (Figure 5I) and CFA (Figure 5J) rats. In both models, we found that 16 daily intravenous injections were needed to achieve complete alleviation of pain. Remarkably, however, after interruption of the treatment, the analgesic effects lasted for 9 days. As observed with intrathecal injections (Figure 5, E–H), RGS scores and ratios obtained during the SWB test of groups of SNL (Figure 5, K and L, respectively) or CFA (Figure 5, M and N, respectively) rats indicated also that intravenous injections of FXYD2-LASO-Gapmer in rats with neuropathic and inflammatory pain significantly alleviated their spontaneous pain symptoms.

Altogether, these data demonstrate the advantages of using FXYD2-LASO-Gapmer in a therapeutic protocol to treat neuropathic or inflammatory pain in the long term, consisting of daily intrathecal or intravenous injections of 2 μg or 40 mg/kg, respectively, of the molecule during 10–16 days, followed by a single injection, at the same concentration, every 8–10 days, that might be easily adaptable to human patients.

## Discussion

In the present study, we report the characterization and the validation of a new antisense-based therapy to efficiently treat chronic peripheral pain disorders that could be directly adaptable to human patients. Indeed, by screening target sequences in the human *FXYD2* mRNA favorable for antisense inhibition, we notably identified a region of 20 nucleotides 100% identical in rat and human. One originality and strength of this study was to use this human evolutionarily-conserved sequence as basis for the design of therapeutic molecules consisting of chemically modified FXYD2-ASOs (in-house-synthesized FXYD2-LASO and FXYD2-LASO-Gapmer) and FXYD2-siRNA (custom-made FXYD2-Accell siRNA). After their validation in HEK293M cells in vitro as potent inhibitors of FXYD2 expression, these antisense molecules were directly tested in rats to (a) evaluate their efficacy to knock down FXYD2 in DRG neurons in vivo, and (b) set up a therapeutic protocol for alleviating peripheral chronic pain induced by nerve injury and inflammation. We established that the most efficient and least invasive strategy for long-lasting pain relief consisted of a 2-phase treatment: a first phase of daily intrathecal or intravenous injections of 2 μg or 40 mg/kg, respectively, of FXYD2-LASO-Gapmer during 10–16 days to stably reverse the chronic pain state; and a second phase of maintenance of the analgesic effects over the very long term achieved by a single intrathecal or intravenous injection of 2 μg or 40 mg/kg, respectively, of FXYD2-LASO-Gapmer every 8–10 days.

Nowadays, increasing numbers of antisense-based therapies are being developed in many domains. Yet the use of ASOs or siRNAs in vivo raises several challenges, such as specific cell delivery, intracellular penetration, or long-term stability. In vitro, these issues can be circumvented by their direct application using transfecting reagents. However, these reagents can exhibit cellular toxicity, which prevents their routine use for drug delivery in vivo. During the past years, several chemical transformations have been developed to overcome these problems (34). In this study, all ASOs were produced with the classic phosphorothioate backbone modifications reported to improve stability and cellular uptake. In our case, however, this modification alone was not sufficient to ensure functional efficacy since FXYD2-ASO was unable to knock down FXYD2 expression whether in DRG neurons in vivo or in vitro in HEK cells without transfecting reagents. In contrast, a lipid modification added to this ASO consisting of adjunction of hydrophobic chains (25) made the resultant FXYD2-LASO a potent inhibitor of FXYD2 expression as well as an efficient analgesic molecule without the use of transfecting reagent. The formulation of this FXYD2-LASO was further improved by use of 2′-*O*-2-methoxyethyl sugar modifications, known to enhance stability (34), to generate the so-called FXYD2-LASO-Gapmer. This gapmer turned out to be particularly interesting since it allowed a great reduction in the frequency of intrathecal or intravenous injections to maintain long-lasting analgesia during the second phase of the treatment, from a daily basis using FXYD2-LASO to an 8–10 days basis using the FXYD2-LASO-Gapmer. Intriguingly, weekly intrathecal injections of this gapmer, even at high doses (300 μg), were not as beneficial during the first phase of the treatment, which still required daily injections, though at low doses (2 μg). This seems not due to the great stability of the FXYD2 protein, since we determined that its half-life is in the medium range. In addition, we have demonstrated that the treatment causes a relatively rapid reduction in protein level that precedes the improvement in pain behavior by several days. Therefore, the need to deliver the gapmer daily during the first phase of the treatment probably reflects transient compensatory mechanisms that remain to be deciphered.

In this study, we report 2 efficient modes of delivery for the FXYD2-LASO-Gapmer: intrathecal and intravenous injections. Keeping in mind that these techniques may engender some discomfort for patients, notably during the initial incompressible 10- to 16-day period of daily injections, alternative methods could be envisaged. In particular, it has recently been shown that ASOs can be delivered to DRG by subcutaneous injections (35), which might be thus considered, though a significant increase in the doses injected would certainly be needed to reach comparable analgesic effects. Another recent study has reported the use of recombinant adeno-associated viruses (AAVs) to deliver a dCas9-sgRNA directed against Na_v_1.7 into the DRG for long-term alleviation of pain in mice (36). Although promising and possibly adaptable to knock down *FXYD2*, this approach has the disadvantage of the virtually permanent presence of the AAV in the DRG neurons for years after the treatment. Thus, although more invasive, intrathecal and intravenous administration strategies appear as relevant and feasible methods transposable to human patients. In line with this, intrathecal administration has been recognized for ASO delivery in several clinical trials, including on patients with amyotrophic lateral sclerosis (tofersen) or with Huntington’s disease (tominersen), and approved for the treatment of spinal muscular atrophy with nusinersen (Spinraza) (37, 38). Moreover, an intrathecally injected double-stranded DNA decoy–based drug called brivoligide is under development for the treatment of postoperative pain (39). Interestingly, we and others have validated this method for ASO delivery into DRG neurons in rodents (e.g., refs. 16, 40), while avoiding rapid accumulation, metabolism, and elimination by the liver and kidneys (41). This is particularly interesting since somatosensory neurons of the DRG are the only cells along the somatosensory pathway in which FXYD2 is expressed (14–16) and thus represent a highly specific target cell type for FXYD2 antisense inhibition and alleviation of chronic peripheral pain symptoms (refs. 15, 16, and this study). Finally, it is important to note that a possible alternative method for long-term intrathecal delivery of FXYD2-LASO-Gapmer without repeated manipulations might be the use of implantable pumps. Interestingly, these devices have been used for many years to notably deliver 2 important medications for chronic pain management, ω-conotoxin (ziconotide, or Prialt) and morphine (reviewed in ref. 42).

The calcium channel antagonist ω-conotoxin MVIIA is the current gold standard to treat severe chronic pain in patients for whom intrathecal therapy is warranted, and who are intolerant or refractory to other treatments, such as systemic analgesics, adjunctive therapies, or intrathecal morphine. We have shown that FXYD2-LASO-Gapmers are at least as efficient as ω-conotoxin for pain relief. Both treatments, however, have very different temporal dynamics. Indeed, while ω-conotoxin acts rapidly but during a very short time window (3 hours), FXYD2-LASO-Gapmers act over a very long range (8–10 days), albeit after a latency phase. On the other hand, opioids (morphine, codeine, oxycodone) are currently one of the most potent groups of analgesics used clinically (43). However, such molecules often induce adverse side effects; can lead to tolerance, which necessitates constant dose increases; and can cause addiction. We have shown that the pain relief effect of FXYD2-LASO does not involve the endogenous opioid system and does not seem to lead to tolerance. Indeed, we observed that daily delivery of a low dose (2 μg) of FXYD2-LASO or of FXYD2-Acell siRNA, during approximately 3 weeks in rat models of chronic pain, triggered constant and high analgesic effects throughout the entire period of the treatment without the need of increasing doses. The issue of side effects is a key parameter when considering the efficacy of a treatment. As mentioned above, while efficient in the short term, administration of opioids over the long term can cause adverse side effects that preclude their general utilization for chronic pain management. For this reason, alternative strategies are continuously being explored (29). These notably include the use of repurposed anti-epileptics and antidepressants, although their broad range of neuronal targets in the central nervous system may be contraindicated in some cases (10). Other approaches are dedicated to blocking the expression and/or function of members of different families of ion channels such as Na_v_1.7, Na_v_1.8, Ca_v_3.2, TREK, or ASIC channels, known to be involved in pain processing (36, 44–47). Finally, another recent promising strategy was developed to selectively silence interneuron populations in the dorsal spinal cord that participate in the establishment of a chronic pain state, through the use of synthetic botulinum molecules (48). However, again, most of these strategies potentially have broader impacts than expected not only on neurons of the central nervous system but also on vital organs, owing to the fact that ion channels in particular are largely distributed throughout the organism. The issue of selective cell targeting is therefore crucial. In this context, one important advantage of targeting *FXYD2* resides in its relatively restricted distribution in the body. Indeed, in the nervous system it is restricted to neurons of the DRG and is largely excluded from the brain and the spinal cord. Outside the nervous system, its main expression sites are the kidneys, where it influences ion transport. In line with this, mutations in the human *FXYD2* gene cause hypomagnesemia. Nevertheless, this deficiency can be relatively easily treated by oral or intravenous magnesium supplementations (49). Importantly, in our experimental paradigms, intrathecal administration of low doses of ASOs or siRNA did not trigger a significant reduction of FXYD2 protein levels in kidneys, in contrast to the DRG. Moreover, global evaluation of the health and behavior of FXYD2-LASO–treated rats did not reveal obvious deleterious side effects of the treatment. This suggests that local administration of FXYD2-LASO-Gapmer or FXYD2-Accell siRNA to the DRG by intrathecal injections may have virtually no side effects, while the use of more systemic delivery methods may only cause limited or, at worst, manageable side effects.

The mechanism by which FXYD2 inhibition alleviates chronic pain symptoms may reflect, at least in part, its ability to modulate the activity of the Na,K-ATPase pump in DRG neurons. Indeed, after peripheral nerve lesions or inflammation, FXYD2 has been shown to reduce the activity of this pump and to allow persistent neuronal hyperexcitability notably of the so-called non-peptidergic IB4-positive nociceptive population. In turn, afferent synaptic transmission to central neurons is facilitated, which participates in the establishment of long-lasting mechanical allodynia (15, 16). Consistent with this, in *Fxyd2*-mutant mice, the injury-induced hyperexcitability of the IB4-positive nociceptors is reverted (16). Numerous transcriptomic studies focused on the DRG have also shown that induction of persistent pain is accompanied by a complex temporal sequence of changes in gene expression, depending on the neuronal population and the type of injury (27). This constitutes a molecular signature of a chronic pain state. Interestingly, our transcriptomic analysis of 43 “signature genes” showed that the beneficial impact of the FXYD2-LASO treatment on pain behaviors is not accompanied by a reversal of the molecular changes induced by injury or inflammation. This suggests that FXYD2 knockdown simply blocks the transmission of the aberrant pain signal without deep reversal of the chronic state. This is also supported by the fact that discontinuation of the FXYD2-LASO, FXYD2-LASO-Gapmer, or FXYD2-Accell siRNA treatments led to the resurgence of pain symptoms at some point.

In conclusion, this study establishes the use of stabilized and lipid-modified FXYD2-ASO or siRNA as highly potent therapeutic molecules to treat chronic pain symptoms induced by peripheral nerve injury or inflammation over the long term, with no — or limited — side effects, potentially directly applicable to human patients. It is of note that while the validation of this therapeutic protocol was achieved in rat models by targeting of the only 20-mer stretch in the *FXYD2* mRNA coding region evolutionarily conserved between rat and human, we have also characterized other human-specific FXYD2-ASOs with even higher knockdown properties in HEK cells. This leaves open an interesting opportunity to eventually further improve the efficiency of the treatment when applied to patients.

## Methods

Further information can be found in Supplemental Methods.

### Animals.

All animals were housed on a 12-hour dark/12-hour light cycle with ad libitum access to water and food. Five-week-old female or male Sprague-Dawley rats (Janvier), weighing 200–250 g at the beginning of the experiments, were used.

### Chronic pain models.

The spinal nerve ligation (SNL) model of peripheral neuropathic pain and the compete Freund’s adjuvant (CFA) model of chronic inflammatory pain were used. All surgical procedures were performed under deep isoflurane anesthesia. The SNL procedure was performed as described previously (26). Briefly, the L6 transverse process was removed to expose the L4 and L5 spinal nerves. The L5 spinal nerve was then isolated and tightly ligated with 6.0 silk thread. To obtain sham animals, the gesture of exposing the nerve was carried out but no ligature was performed. The model of CFA-induced pain (50) was used for assessing chronic inflammatory pain. Briefly, under isoflurane anesthesia, an intraplantar injection (50 μL) of a solution of 1 mg of *Mycobacterium tuberculosis* (Sigma-Aldrich) per mL was performed in the left hind paw of animals. Control animals were obtained by injection of 50 μL of saline solution (NaCl 0.9%).

### Commercial Accell siRNA and synthesis of ASO, lipid-conjugated LASO, and 2′-MOE–modified LASO gapmer.

An Accell siRNA directed against rat-human *FXYD2* mRNA for in vivo use, called FXYD2-Accell siRNA, was purchased from Horizon Discovery Ltd. The sense sequence was 5′-AAGAUUCCGCUGUGGGGGC(UU)-3′. The Accell nontargeting in vivo siRNA, called control-Accell siRNA (D-001910-01, Horizon Discovery Ltd.), was used as a negative control.

### Synthesis of ASOs, LASOs, and LASO-Gapmers.

Oligonucleotides were synthesized using the conventional phosphoramidite chemistry on automated synthesizers at scales from 1 to 40 μmol. The H8 DNA synthesizer (K&A Labogeraete) was used to synthesize 1 μmol of oligonucleotides for preliminary in vitro studies on controlled-pore glass solid support (Link Technologies). The AKTA Oligopilot 10 (GE Healthcare) was used to synthesize larger-scale sequences ranging from 25 to 40 μmol. These batches synthesized on polystyrene solid support (GE Healthcare) were used for in vivo studies. DNA and 2′-MOE monomers and synthesis reagents originated from Glen Research. The lipid oligonucleotides were modified at the 5′ end with a double-chain C16 ketal nucleolipid phosphoramidite synthesized in the laboratory as described previously (51). All oligonucleotides were synthesized with phosphorothioate chemistry as the inter-nucleoside link, purified on an HPLC column, and characterized by electrospray ionization mass spectrometry carried out on a Thermo Fisher Scientific Q-Exactive (Supplemental Figure 6, A–E, and Supplemental Table 1). Cleavage and deprotection were achieved in 28% ammonia for 4 hours at 55°C.

A nontargeting ASO, LASO, and LASO-Gapmer (sense sequence: 5′-CGTGTAGGTACGGCAGATC-3′) were used as a negative control and called control-ASO, -LASO, and -LASO-Gapmer. We designed 29 different ASOs directed against human *FXYD2* mRNA; the sequences are shown in Supplemental Figure 1A.

### Intrathecal or intravenous ASO, LASO, Accell siRNA, or ω-conotoxin MVIIA and subcutaneous naloxone injections in rats.

SNL-operated rats were tested to confirm mechanical hypersensitivity and were then injected daily intrathecally with 2 μg of control-Accell siRNA, -ASO, -LASO, or -LASO-Gapmer; or FXYD2-Accell siRNA, -ASO, -LASO, or -LASO-Gapmer in 20 μL of 5% glucose solution in water under brief isoflurane anesthesia (40). For CFA-injected rats, 2 μg of control- or FXYD2-LASO or control- or FXYD2-LASO-Gapmer in 20 μL of 5% glucose solution in water was injected daily intrathecally from day 3 after CFA injection. For daily intravenous injections, 40 mg/kg of control- or FXYD2-LASO-Gapmer in 200 μL of 5% glucose solution in water was given under brief isoflurane anesthesia to SNL-operated and CFA-injected rats. Intraplantar injection of naloxone (1453005, Sigma-Aldrich; 1 mg/kg) was performed as described previously (30). ω-Conotoxin MVIIA was purchased from Sigma-Aldrich (C1182), and a dose of 100 pmol was intrathecally injected as described in ref. 31.

### Behavioral testing on rats.

Sprague-Dawley female or male rats were housed 2 per cage under standard conditions of light and temperature. Commercial chow pellets and tap water were available ad libitum. After arrival, animals were left to become accustomed to the colony room for 4 days. To avoid stress resulting from the experimental conditions, analyses were performed by the same experimenter in quiet conditions in a test room close to the colony room. For 2 weeks before the experiments, animals were weighed daily, handled gently for 5 minutes, and placed in the test room for 1 hour, where they were left to become accustomed to the nociceptive apparatus. Mechanical allodynia and mechanical hyperalgesia were evaluated days before and the day of surgery (day 0) and once daily after surgery. For mechanical allodynia, we performed the von Frey test, as previously described (16). For mechanical hyperalgesia, nociceptive thresholds in handheld rats were determined with the Randall-Selitto paw pressure test, as previously described (52), using the Basile analgesimeter (Apelex; stylus tip diameter, 1 mm). Briefly, a constantly increasing pressure was applied to the injured hind paw until the rat squeaked. A 600 g cutoff value was determined to prevent tissue damage. Mobility and anxiety-like behavior were also evaluated with the open field experiment. The rats were placed in the center of the open field arena (100 × 100 cm), and movements were recorded with infrared captors during 10 minutes. The time spent in the center (central 60 × 60 cm) or in the periphery (external 20 cm) was analyzed with EthoVision XT 15 software, and the total distance traveled by each group was recorded. Two non-reflexive behavioral tests were used to evaluate spontaneous pain: the Rat Grimace Scale (RGS) and the Static Weight Bearing (SWB) test. The RGS test was performed as described previously (53). Four action units of the RGS were observed: orbital tightening, nose/cheek flattening, ear changes, and whisker changes. Two different scorers assigned a value of 0, 1, or 2 for each of the 4 RGS action units (0: action unit was absent; 1: moderate appearance of the action unit; 2: obvious appearance of the action unit) to previously randomized animals. The RGS score corresponds to the average value obtained for each action unit. The SWB testing system (Bioseb) was used for incapacitance testing. Rats were placed in the testing box and allowed to acclimatize before recording for approximately 5 minutes. The 2 hind paws were placed on 2 independent platforms, and the force exerted by each hind paw was measured in grams. Triplicates were performed for each hind paw and for each animal. The weight ratio between the left and the right hind paw was calculated.

Note that in SNL and CFA rat models used in all experiments, the left hind paw was lesioned.

Von Frey, Randall-Selitto, open field, RGS, and SWB tests were performed on 6–9 animals for each experimental condition.

### In vitro FXYD2 protein knockdown experiments with ASO.

Control-ASO and 29 different ASOs directed against human *FXYD2* mRNA were tested in vitro in HEK293M cells. HEK293M cells were maintained in DMEM Glutamax (Invitrogen) supplemented with antibiotics (penicillin 50 U/mL, streptomycin 20 μg/mL) and 10% heat-inactivated FCS. Cells were plated at a density of 50% and treated after 1 day with the indicated ASO for 2 days. Lipofectamine 2000 (Invitrogen), a cationic lipid, was used to increase ASO uptake into the cells. Cells were treated with 100 nM of ASO after a preincubation for 20 minutes with Lipofectamine 2000 diluted at 1:1,000 in serum-free OPTI-MEM (Life Technologies). After 4 hours, the medium was replaced with standard culture medium described above. Control- and FXYD2-LASO were tested in vitro in HEK293M cells as described above but without the use of Lipofectamine 2000.

### In vivo FXYD2 protein knockdown experiments with LASO.

Zero, 0.5, 2, or 4 μg of FXYD2-LASO in 20 μL of 5% glucose solution was injected daily intrathecally during 14 days. For each concentration of FXYD2-LASO, 3 rats were injected. Rats received an intraperitoneal injection of pentobarbital and were transcardially perfused with PBS. Lumbar DRGs (L4 and L5) were dissected and stored at –80°C.

### Western blot.

Cells or tissues were mechanically homogenized at 4°C in NP40 buffer (1% NP40, 150 mM NaCl; 50 mM Tris-HCl, pH 7.5, and protease inhibitor). Lysates were clarified for 10 minutes at 4°C at 12,000*g*. After protein quantification using a BCA kit (Thermo Fisher Scientific, France), lysates were run on SDS-PAGE and transferred to nitrocellulose membrane. Rabbit anti–C-terminal FXYD2 antibody (provided by S. Karlish, Weizmann Institute of Science, Rehovoth, Israel; diluted 1:2,000) shows the presence of the FXYD2 isoforms a and b. Mouse anti-actin (A3853, Sigma-Aldrich; diluted 1:1,000) antibody was used for normalization. After incubation with primary and fluorescent IRDye secondary antibodies (anti-mouse IRDye 680BD, 926-68072, and anti-rabbit IRDye 800CW, 926-32213, LI-COR Biosciences; diluted 1:15,000), immunodetection was performed using Odyssey CLx Imager (LI-COR Biosciences). Quantifications were done with Image Studio Lite software (LI-COR Biosciences).

### Statistics.

For Western blot experiments and RGS and SWB tests, statistical analyses were performed using 2-tailed unpaired Student’s *t* test or 1-way ANOVA followed by a post hoc Bonferroni’s test. For behavioral studies, von Frey and Randall-Selitto tests and group and time effects were validated by 2-way ANOVAs for repeated measurements. When ANOVAs showed a significant effect, Bonferroni post hoc test was used to determine the significance of the differences. *P* values less than 0.05 (*), 0.01 (**), 0.001 (***), and 0.0001 (****) were considered as statistically significant. All data presented are means ± SEM. For NanoString nCounter analysis, Pearson correlation between sample pairs was plotted as heatmaps, in order to visualize the grouping of similar samples, using nSolver Analysis Software v4.0 (NanoString Technologies).

### Study approval.

All animal experiments were approved by the French Ministry of Research (authorizations 17923 and 32862) and performed according to the guidelines of the International Association for the Study of Pain.

## Author contributions

PC, AP, and SV conceived and designed the experiments. AD, NS, VB, SB, BV, and SV performed the experiments. AD, NS, PB, PC, AP, and SV analyzed the data. AD, NS, SB, BV, CR, and PB developed methodology and contributed materials. PC, AP, and SV wrote the manuscript.

## Supplementary Material

Supplemental data

## Version 1 05/08/2023

Electronic publication
