# Supplementary material for: FXYD2 antisense oligonucleotide provides an efficient approach for long-lasting relief of chronic peripheral pain
Source: JCI Insight. 2023 May 8;8(9):e161246. doi: 10.1172/jci.insight.161246 (PMC10243811; doi:10.1172/jci.insight.161246)
Supplement: Supplemental data [file jciinsight-8-161246-s045.pdf]

Supplemental Material

FXYD2 Antisense Oligonucleotide Provides an Efficient Approach for Long-lasting Relief of Chronic Peripheral Pain

Authors: Alexandre Derre[1]†, Noelian Soler[1]†, Valentine Billoux[1], Sebastien Benizri[2], Brune Vialet[2], Cyril Rivat[1], Philippe Barthelemy[2], Patrick Carroll[1]‡, Alexandre Pattyn[1]‡, Stephanie Venteo[1]*

Methods

Purification and characterization of oligonucleotides. Crude oligonucleotide sequences without the 5’ lipid moiety were directly dialyzed after synthesis. Dialysis of the samples was performed against a 9% NaCl solution followed by distilled water. The membrane used was 2kD MWCO (molecular weight cut off) Spectra Por 6 (Spectrum). Analysis of these sequences ASO75 and Scramble was performed after dialysis using reversed-phase HPLC on a Hitachi LaChrom EliteHPLC System with gradient standard protocol. The C18 column (Waters, Xbridge Oligonucleotide BEH, 130 Å, 2.5 µm, 4.6 mm x 50 mm) was used. A gradient was applied to elute the molecules, starting with 100% phase A and decreasing to 70% phase A in 10 min. The column was kept for 2 min at 70% A, then returned to 100% A in 2 min and maintained for 1 min at 100% A. Mobile phase A solution contained 100 mM triethylammonium acetate (TEAA) equilibrated at pH 7 and 5% acetonitrile (ACN) and phase B contained 20 mM TEAA and 80% ACN. The flow rate was fixed at 2.8 mL/min. The injection volume of samples was 5 to 25 µL. All oligonucleotides were detected at a wavelength of 260 nm. The lipid-oligonucleotides DNA and gapmer 2’MOE sequences were purified on a preparative C4-reverse phase HPLC column (Waters, Xbridge Protein BEH, 300 Å, 5 µm, 30 mm x 50 mm). Using the same eluting phases, a 4 min gradient was applied. The columns starting with 100% A is shifted into 100% B in 2 min, stays for 1 min at 100% B, then returns to 100% A at 3.2 min. The flow rate was fixed at 56 mL/min. After concentration of the samples, the oligonucleotides underwent a dialysis step described earlier. Purified lipid oligonucleotides were analyzed with an HPLC Nucleosil C4 column (4 × 250 mm, 5 µm) from Macherey-Nagel. A linear gradient was programmed from 100% A to 100% B in 10 min. A mobile phase composed of 100% B was kept during 2 min and the column was then equilibrated during 5 min with 100% A before the following run. The flow rate was fixed at 1.0 mL/min. All samples were characterized by ESI mass spectrometry carried out on a Thermo Fisher Q-

Exactive (Figure S6A-E, Table S1). Oligonucleotide samples were prepared using 3.5 kD vivacon membrane (Sartorius) for dialysis against 50 mM ammonium acetate (Sigma-Aldrich). All oligonucleotides samples were lyophilized and stored at -20°C.

Cycloheximide chase. FXYD2 protein half-life was determined by the cycloheximide chase method. HEK293M cells were cultured as described above and transfected with 1,5 µg/ml of human *FXYD2* plasmid (RC205076, Origene) using Lipofectamine 2000 as transfection agent. 24 h after transfection, cells were incubated with cycloheximide (50 µg/ml, Sigma-Aldrich, Ref. C4859) for various time periods (0 h, 0.5 h, 1 h, 3 h, 6 h, 9 h, 12 h, 24 h or 48 h). Cells were harvested for protein extraction at different time intervals.

Nanostring nCounter analysis. Total RNA was extracted and purified from DRGs L5 using the RNeasy Mini Kit (Qiagen) and quantified on a NanoDrop 2000 (Thermo Fisher Scientific) according to the manufacturers' instructions. For each sample, 100 ng of total RNA were subjected to nCounter™ SPRINT (NanoString Technologies) analysis. A custom CodeSet of capture and reporter probes were designed and synthesized by NanoString nCounter™ technologies to target 43 selected genes (Figure S4). Each target gene of interest was detected using a pair of reporter and capture probes that together target a continuous 100 nucleotide sequence. Hybridization between target mRNA and reporter-capture probe pairs was performed at 65°C for 17 h using mastercycler nexus GSX1 (Eppendorf) ; detection and scanning followed the manufacturer's recommendations. Quality control and normalization of the raw data were performed using nSolver Analysis Software v4.0 (NanoString Technologies). Normalization was performed using the geometric mean of the four stable transcripts (Hprt1, Polr2j, Ddx48 and Rpl13a). 8 negative controls were used to determine the background threshold based on calculating the average count of these negative controls. Genes that had counts above the

threshold were considered as detected genes. A background threshold of 20 was selected, to which all raw counts at or below were set.

Figures

A

ASO	Position	5'-3' Sequence	% GC
112	112-131	TCATAGTAGAACGGGTCCAC	50
113	113-132	GTCATAGTAGAACGGGTCCA	50
114	114-133	AGTCATAGTAGAACGGGTCC	50
115	115-134	TAGTCATAGTAGAACGGGTC	45
154	154-173	AGTCCAGCGAAGATCAGGCC	60
155	155-174	CAGTCCAGCGAAGATCAGGC	60
156	156-175	CCAGTCCAGCGAAGATCAGG	60
157	157-176	GCCAGTCCAGCGAAGATCAG	60
158	158-177	GGCCAGTCCAGCGAAGATCA	60
208	208-227	CCCCCACAGCGGAATCTTCT	60
210	210-229	TGCCCCCACAGCGGAATCTT	60
211	211-230	TTGCCCCCACAGCGGAATCT	60
212	212-231	ATTGCCCCCACAGCGGAATC	60
213	213-232	TATTGCCCCCACAGCGGAAT	55
214	214-233	TTATTGCCCCCACAGCGGAA	55
215	215-234	CTTATTGCCCCCACAGCGGA	60
216	216-235	TCTTATTGCCCCCACAGCGG	60
217	217-236	TTCTTATTGCCCCCACAGCG	55
218	218-237	CTTCTTATTGCCCCCACAGC	55
219	219-238	GCTTCTTATTGCCCCCACAG	55
221	221-240	GCGCTTCTTATTGCCCCCAC	60
225	225-244	GCCTGCGCTTCTTATTGCCC	60
231	231-250	TGATTTGCCTGCGCTTCTTA	45
240	240-259	CATCTTCATTGATTTGCCTG	40
244	244-263	GGCTCATCTTCATTGATTTG	40
245	245-264	CGGCTCATCTTCATTGATTT	40
246	246-265	ACGGCTCATCTTCATTGATT	40
247	247-266	TACGGCTCATCTTCATTGAT	40
248	248-267	TTACGGCTCATCTTCATTGA	40

B

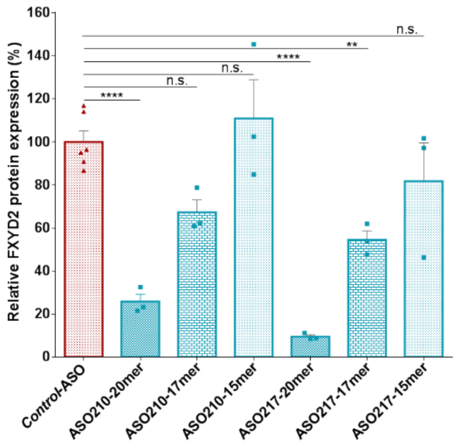

Figure S1. Identification of oligonucleotide sequences with potential FXYD2 knock-down properties. Sequences of 29 antisense oligonucleotides tested for their capacity to diminish FXYD2 protein levels in HEK293M cells. The numbers represent the starting base of each 20-mer oligonucleotide tested (**A**). Quantification of FXYD2 protein levels by Western blot of extracts of HEK293M cells after transfection by ASO210 and ASO217 with a length of 15, 17 and 20 nucleotides each (**B**). The levels of FXYD2 protein were normalized to actin protein. Data are represented as means ± s.e.m., n=3 replicates. One-way analysis of variance (ANOVA) and post-hoc Bonferroni's test, n.s.=non-significant; **=p<0.01 and ****=p<0.0001.

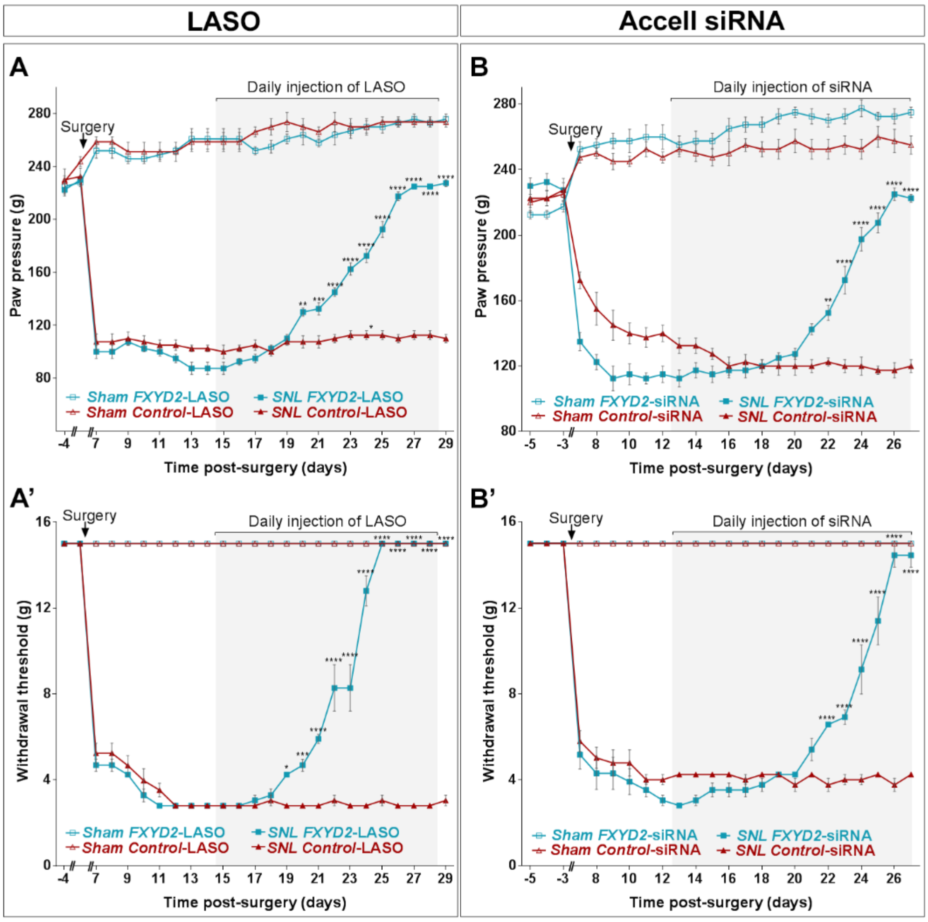

Figure S2. FXYD2- and control-LASO or FXYD2- and control-Accell siRNA had also no effect on Sham-rats behavior. After induction of mechanical hypersensitivity by SNL, animals were intrathecally injected daily with FXYD2- or control-LASO (**A**, **A'**) and FXYD2- or control-Accell siRNA (**B**, **B'**). FXYD2-LASO and -Accell siRNA, but not control-LASO and -Accell siRNA, reduced mechanical hypersensitivity in the Randall-Selitto paw pressure test (A, B) and von Frey test (A', B'). FXYD2- and control-LASO or -Accell siRNA had no effect on Sham-rats behavior (A-B'). Means ± s.e.m. of data from 9 animals. Two-way analysis of variance (ANOVA) and post-hoc Bonferroni's test, *=p<0.05, **=p<0.01, ***=p<0.001 and ****=p<0.0001.

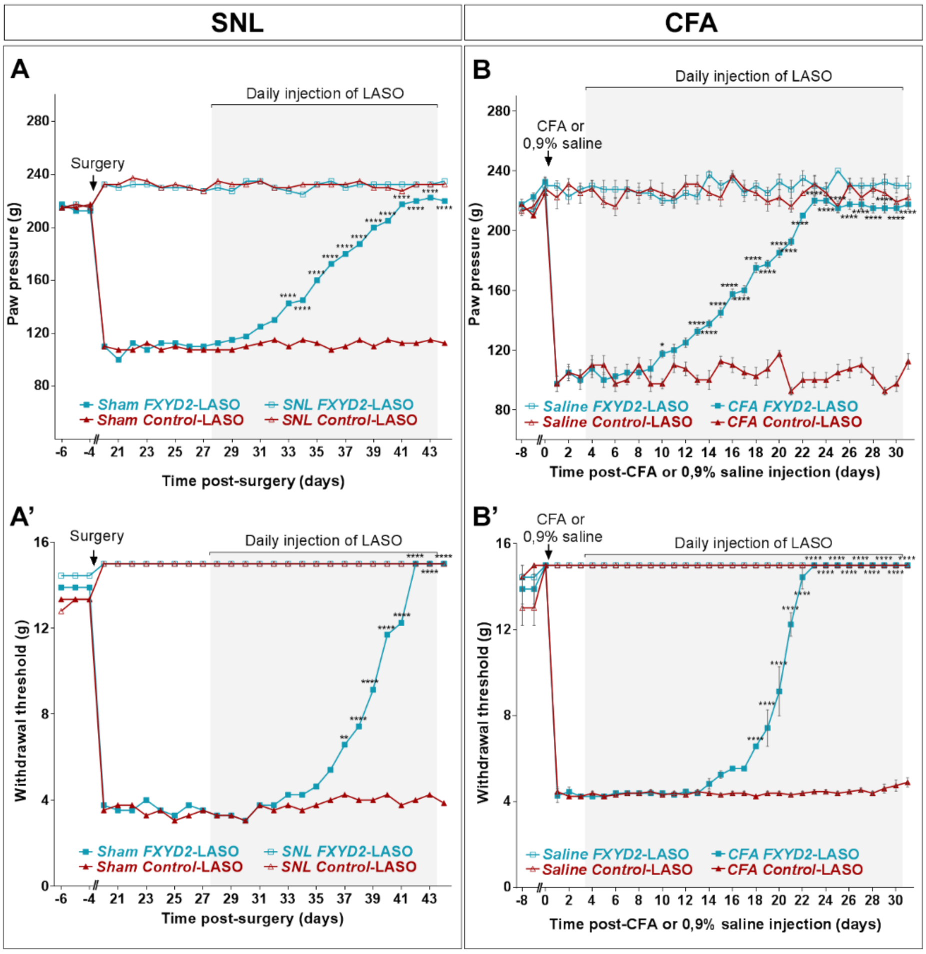

Figure S3. FXYD2-LASO has the same effect on neuropathic or inflammatory pain in female rats as on males. After induction of mechanical hypersensitivity by SNL (**A**, **A'**) or CFA (**B**, **B'**), female rats were intrathecally injected daily with FXYD2- or control-LASO. Similar to results obtained for male rats in Figure 3, female rats displayed reduced mechanical hypersensitivity in the Randall-Selitto paw pressure test (A, B) and von Frey test (A', B'). FXYD2- and control-LASO had no effect on Sham (A, A') or Saline (B, B')-rats behavior. Means ± s.e.m. of data from 6 animals. Two-way analysis of variance (ANOVA) and post-hoc Bonferroni's test, *=p<0.05, **=p<0.01 and ****=p<0.0001.

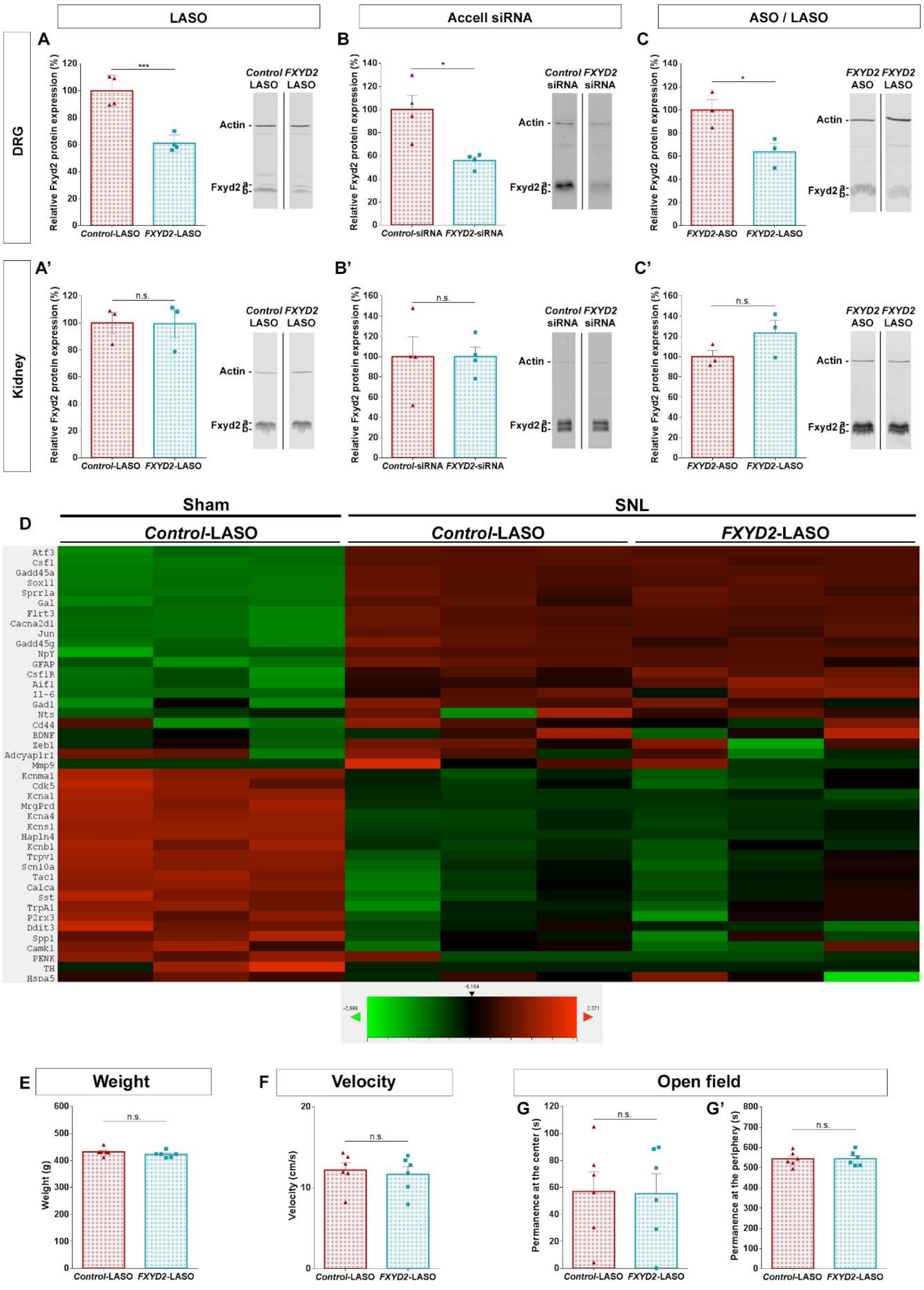

Figure S4. Intrathecally injected FXYD2-LASO has a local action in DRG, does not induce changes in gene expression and does not appear to effect general health and

behavior in naive rats. FXYD2 protein levels were quantified by Western blot for Lumbar DRGs (L4 and L5) (**A**, **B** and **C**) and kidney (**A'**, **B'** and **C'**) at the end of each in vivo behavior experiment with FXYD2- or control-Accell siRNA (Figure 3 A, B), FXYD2- or control-LASO (Figure 3 C, D) and FXYD2-LASO or -ASO (Figure 3 E, F). The levels of FXYD2 protein were normalized to actin protein. Lanes were transferred onto the same membrane but were noncontiguous. (**D**) Heatmap representing dysregulated genes in ipsilateral DRG L5 from 3 control-LASO treated Sham-animals, 3 control-LASO treated SNL animals and 3 FXYD2-LASO treated SNL animals. (**E**) No differences were observed in animal weights in control-LASO or FXYD2-LASO treatment regimes. Open field behavioral test demonstrating that there is no difference in velocity (**F**) and in permanence at the center (**G**) or at the periphery (**G'**) of the arena suggesting an absence of effect upon weight, general locomotor activity and of development of anxiety, respectively, after FXYD2-LASO treatment. Means ± s.e.m. of data from 3 to 4 animals (A-C') and from 6 animals (E-G'). Two-tailed unpaired Student's *t*-test (A-C'; E-G'), n.s.=non-significant; *=$p<0.05$ and ***=$p<0.001$.

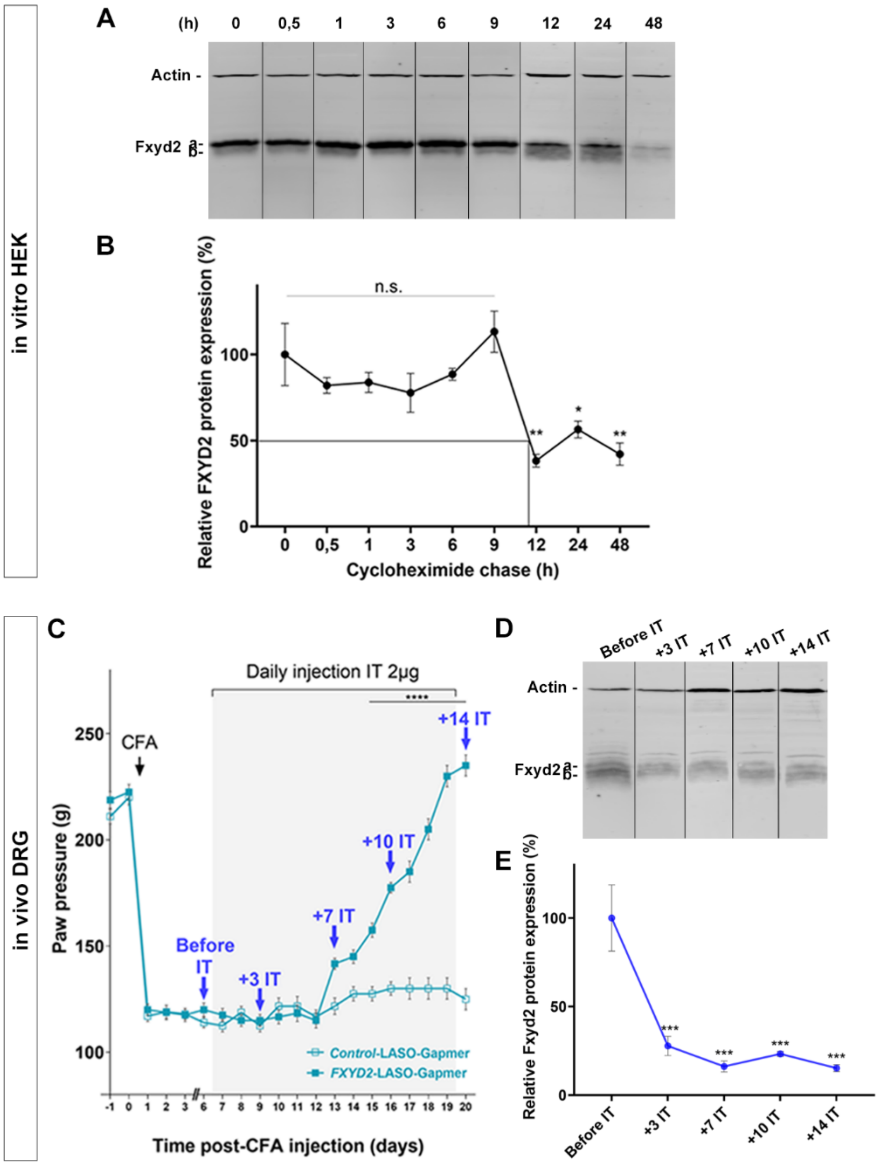

Figure S5. Half-life of FXYD2 protein in vitro and temporal dynamic of FXYD2 knockdown by LASO-Gapmers in vivo. (**A**, **B**) FXYD2 protein half-life was determined by the cycloheximide chase method. HEK293M cells were treated with 50 µg/ml cycloheximide and were harvested at 0 h, 0.5 h, 1 h, 3 h, 6 h, 9 h, 12 h, 24 h or 48 h after drug treatment. Cell lysates were separated on SDS-PAGE and transferred to nitrocellulose membrane. FXYD2 protein levels were quantified by Western blot (A). The levels of FXYD2 protein were normalized to actin protein. FXYD2 levels were plotted as a percentage of the control (0 h) (B). (**C-E**) Mechanical hypersensitivity was induced by intraplantar injection of CFA. Daily intrathecal injections (IT) of 2 µg FXYD2- or control-LASO-Gapmer during 14 days alleviated

pain related behavior (Randall-Selitto paw pressure test) (C). FXYD2 protein levels were quantified by Western blot for Lumbar DRGs (L4 and L5) (D) at various time (indicated in blue in C), before IT, after 3 IT, after 7 IT, after 10 IT and at the end of in vivo behavior experiment after 14 IT. The levels of FXYD2 protein were normalized to actin protein. FXYD2 levels were plotted as a percentage of the control (before IT) (E). Lanes were transferred onto the same membrane but were noncontiguous (A, D). Data are represented as means ± s.e.m. of data from 3 replicates (A, B), 6 animals (C) and 3 animals (D, E). Two-way (C) and one-way (B, E) ANOVA and Bonferroni's test, n.s.=non-significant; *=p<0.1; **=p<0.01; ***=p<0.001 and ****=p<0.0001.

A Calc M: 7093.120
Exp M: 7093.054
ΔM: 9.3 ppm

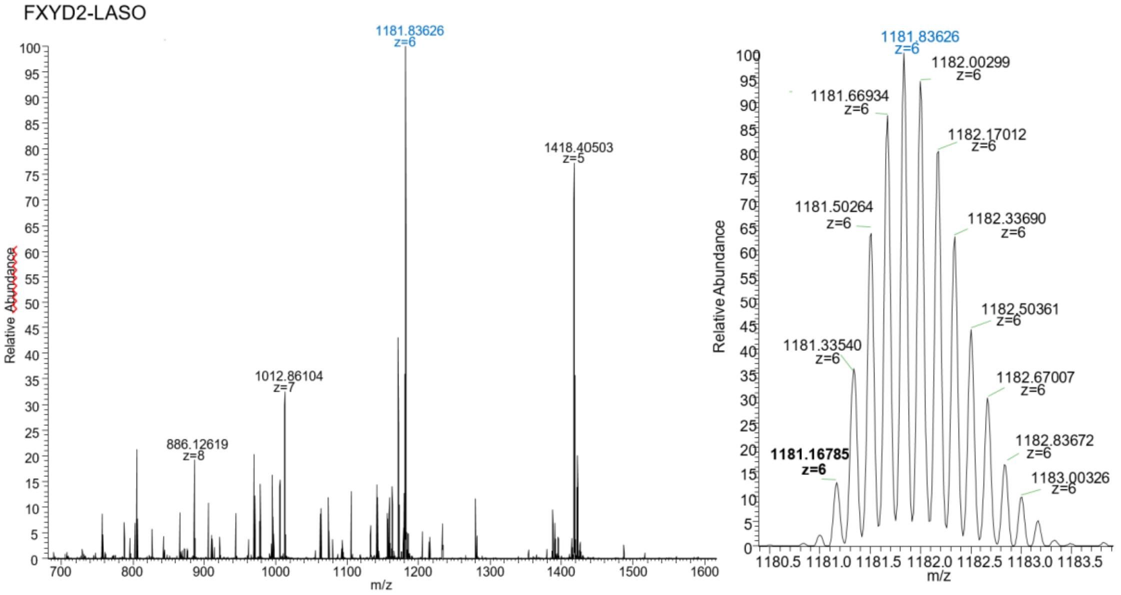

B Calc M: 7889.500
Exp M: 7889.460
ΔM: 5.1 ppm

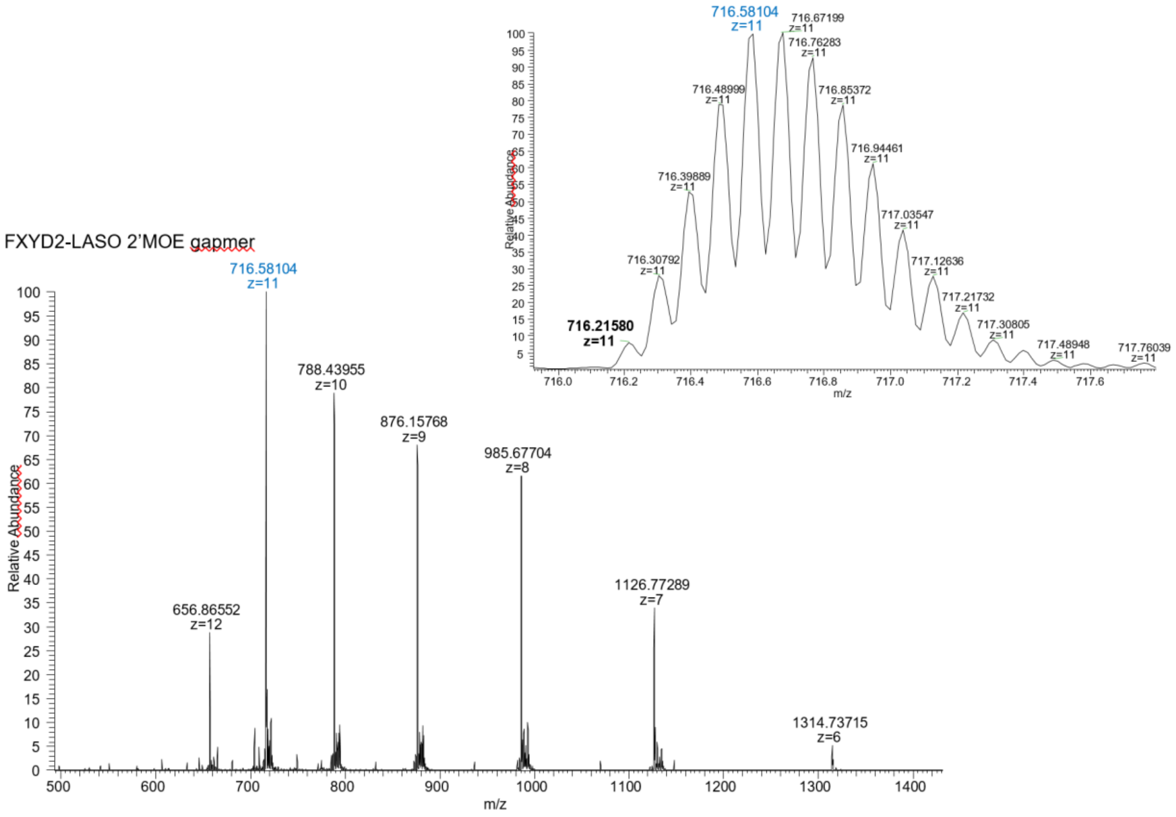

C Calc M : 6153.594
Exp M: 6153.528
ΔM: 10.7 ppm

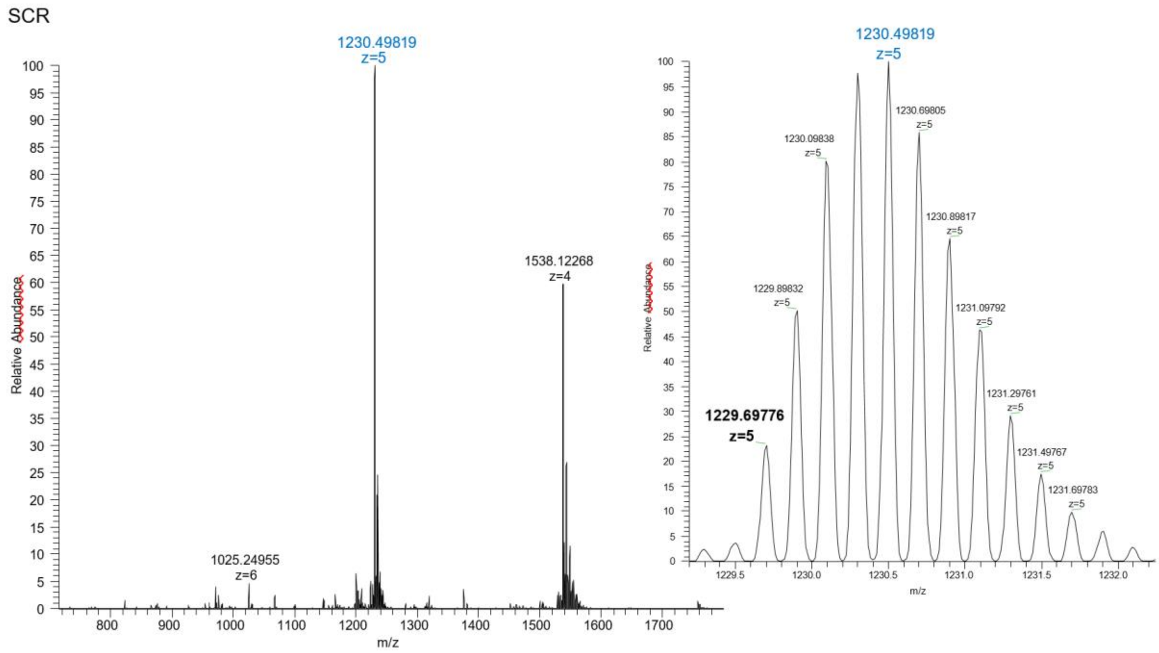

D Calc M: 6908.066
Exp M: 6907.974
ΔM: 13.3 ppm

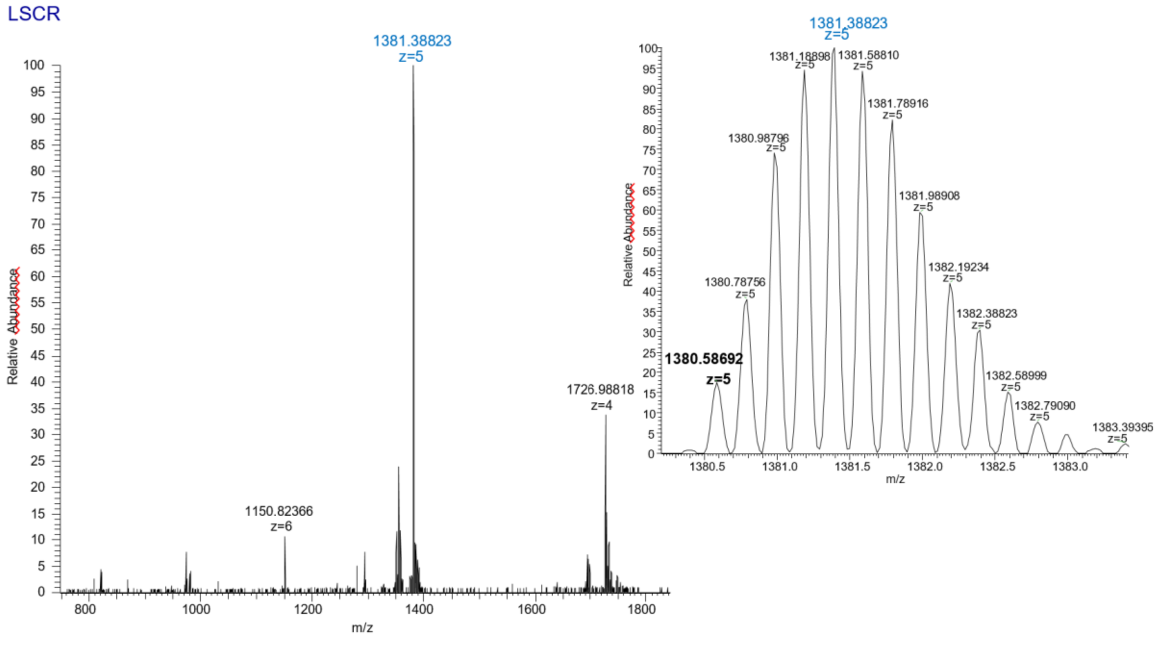

E Calc M: 7676.465
Exp M: 7676.421
ΔM: 5.7 ppm

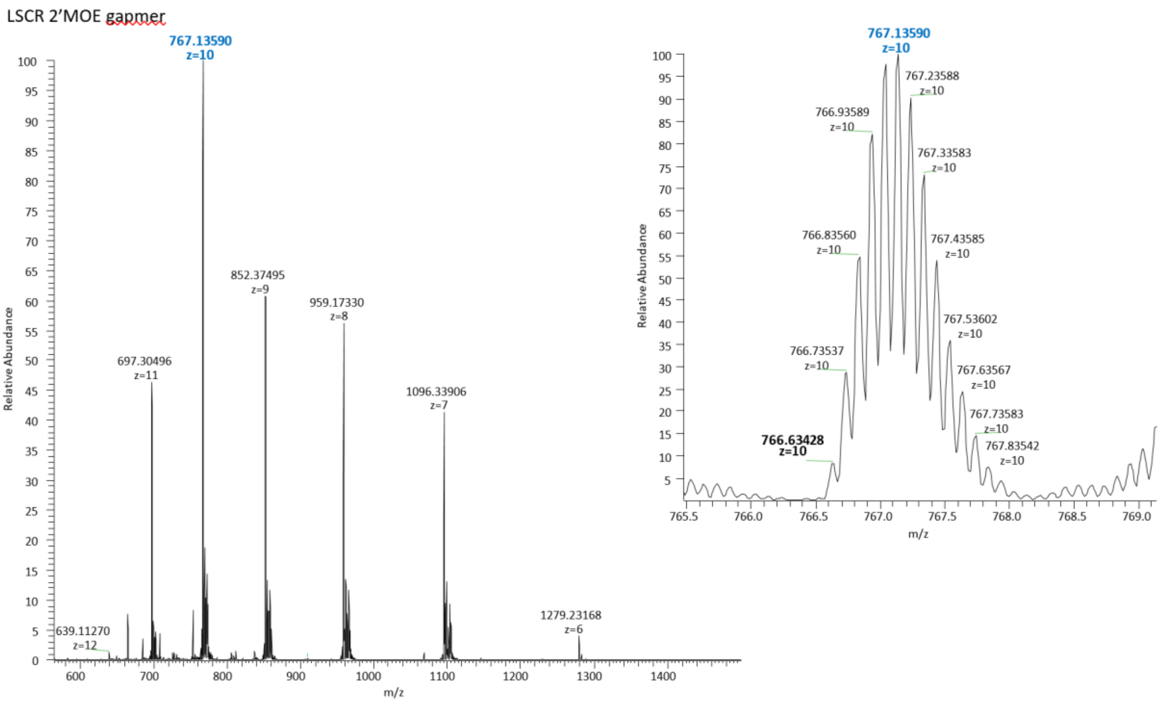

Figure S6. Mass spectrometry spectra. Mass spectrometry spectra of modified oligonucleotides (electrospray ionization, ESI mode), FXYD2-LASO (**A**), FXYD2-LASO 2'MOE gapmer (**B**), SCR (**C**), LSCR (**D**) and LSCR 2'MOE gapmer (**E**).

Name of oligonucleotide sequence	M calculated	M experimental
**FXYD2-LASO**	7093.120	7093.054
**FXYD2-LASO 2'MOE Gapmer**	7889.500	7889.460
**SCR (Scramble)**	6153.594	6153.528
**LSCR (Lipid Scramble)**	6908.066	6907.974
**LSCR 2'MOE Gapmer (Lipid Gapmer Scramble)**	7676.465	7676.421

Supplementary Table 1. Mass spectrometry. Mass spectrometry data (calculated and experimental) of modified oligonucleotides obtained by mass spectrometry in electrospray ionization (ESI) mode.
